# ASPIS Academy: A blueprint for building early-career researcher networks to advance NAM-based risk assessment

**DOI:** 10.1016/j.namjnl.2026.100124

**Published:** 2026-08-07

**Authors:** Luiz Ladeira, Ruben Martinez, Gaëlle Hayot, Barira Islam, Christina H.J. Veltman, Julen Sanz-Serrano, Hiba Khalidi, Rita Ortega-Vallbona, Marie Corradi, Eike Cöllen, A. Melina Steinbach, Klára Gabrišová, Peter Pôbiš, Devon Barnes, Shaleen Glasgow, Marije Niemeijer, Julia Dominika Zajac, Agata Ormanin-Lewandowska, Martijn J. Moné, Bob van de Water, John K. Colbourne, Mathieu Vinken, Silvia Tangianu, Helena Kandarova, Giorgia Pallocca, François Busquet, Eliska Kuchovska

**Affiliations:** a Biomechanics Research Unit, GIGA Institute, University of Liège, Belgium; b Leitat technological center, Spain; c Karlsruhe Institute of Technology, Germany; d Certara Predictive Technologies, UK; e Centre for Health Protection, National Institute for Public Health and the Environment (RIVM), Bilthoven, the Netherlands; f Division of Cell Systems and Drug Safety, Leiden Academic Centre for Drug Research, Leiden University, Leiden, the Netherlands; g Vrije Universiteit Brussel, Belgium; h ProtoQSAR SL, Spain; i HU - University of Applied Sciences Utrecht, the Netherlands; j University of Konstanz, Germany; k German Federal Institute for Risk Assessment (BfR), Germany; l Institute of Experimental Pharmacology & Toxicology, Centre of Experimental Medicine, Slovak Academy of Sciences, Slovakia; m Utrecht University, the Netherlands; n Centre for Environmental Research and Justice, and School of Biosciences, University of Birmingham, UK; o Center for Alternatives to Animal Testing in Europe, Konstanz, Germany; p Humane World for Animals Europe, Belgium; q ALTERTOX, Belgium; r IUF - Leibniz Research Institute for Environmental Medicine, Germany

**Keywords:** Early-career researchers, New approach methodologies, Chemical risk assessment, Scientific networking, Training and mentoring

## Abstract

Chemical risk assessment is moving away from traditional animal-based testing toward New Approach Methodologies (NAMs) that are more human-relevant, efficient, and aligned with the replacement, reduction, and refinement of animal use. This transition depends not only on new methods and technologies, but also on the researchers who will develop, apply, and promote them. Early-career researchers (ECRs) are central to this effort, yet they often work within fragmented professional networks and have limited opportunities for mentoring, visibility, and leadership.

The ASPIS Academy (AA) was established within the ASPIS cluster, comprising the EU Horizon 2020-funded projects ONTOX, RISK-HUNT3R, and PrecisionTox, to support ECRs working on NAM-based chemical risk assessment. Built and coordinated by ECRs with support from senior advisors, AA evolved from an initial idea into an operational network offering training, mentoring, mirroring, twinning, communication, dissemination, and collaboration activities. Its development was shaped not only by strategic planning, but also by the practical realities of voluntary coordination and the changing needs of ECRs across large multidisciplinary consortia.

This manuscript presents the establishment, growth, and legacy planning of AA as a case study for building ECR networks in collaborative research environments. We describe the decisions, challenges, lessons learned, and activities that helped the network become useful to its community and provide a practical blueprint for other consortia seeking to support the next generation of NAM scientists. The AA experience demonstrates that empowering ECRs to lead can strengthen scientific communities and accelerate the uptake of NAMs.

## Highlights

• Blueprint for establishing early-career researcher (ECR) networks.
• ECR-led model progressing to operational maturity within 2 years.
• Integrated programs: training, mentoring, mirroring, twinning, and communication.
• Cross-network collaboration accelerates NAM uptake and visibility.
• Supports 3Rs through capacity building in next-generation risk assessment.

## Graphical abstract

**Figure:**
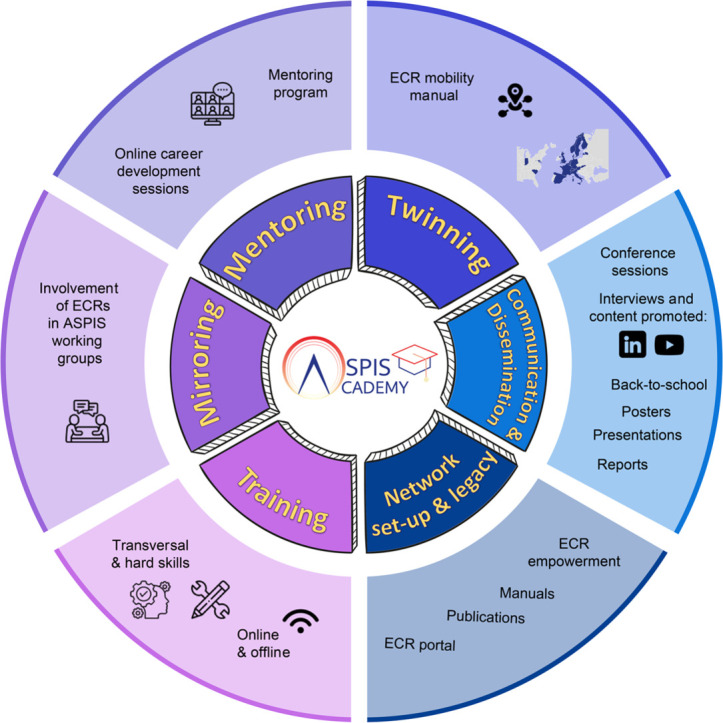

## 1 Introduction

Safety assessment of chemicals is undergoing a major transition from traditional animal-based testing toward New Approach Methodologies (NAMs), driven by ethical concerns, scientific limitations, low throughput, and the need for more human-relevant and mechanistically informed approaches to hazard identification and risk assessment (Schmeisser et al., 2023; Westmoreland et al., 2022; Rovida et al., 2023; Knight, Hartung, and Rovida, 2023; Maertens et al., 2025). In this context, the development, acceptance, fit-for-purpose validation, and implementation of NAMs are central to 21st-century toxicology and next-generation risk assessment (NGRA) (Hartung, 2009; Schmeisser et al., 2023; National Research Council, 2007; van der Zalm et al., 2022). By integrating approaches such as in vitro (Koch et al., 2022; Verhoeven et al., 2025; Maerten et al., 2025; Ückert et al., 2025), *in silico* (Bwanya et al., 2025; Berkhout et al., 2025; Veltman et al., 2025; Gadaleta, Garcia de Lomana, et al., 2024; Gadaleta, Serrano-Candelas, et al., 2024; Garcia de Lomana et al., 2025; Ortega-Vallbona et al., 2025; Isbaita et al., 2026), alternative in vivo species (Holsopple et al., 2023; Mesmar et al., 2024; Jimenez‐Gonzalez et al., 2024; Chaturvedi et al., 2025), exposure and kinetic models (Kalyva et al., 2024; Geci et al., 2025), and omics-based methods (Lislien et al., 2025; Meijer et al., 2025; Viant et al., 2024; Shtetinska et al., 2024; Suciu et al., 2023; Kunnen et al., 2024), NAMs contribute to the replacement, reduction, and refinement of animal use while supporting more efficient and biologically meaningful chemical safety assessment within the One Health concept of toxicological risk assessment (Magurany et al., 2023). However, the successful uptake of NAMs depends not only on scientific and technological innovation but also on the people and communities able to develop, integrate, interpret, and apply these methods in practice. Early-career researchers (ECRs) are particularly important in this regard. They are often well-positioned to work across disciplinary boundaries, combine experimental and computational approaches, and adopt emerging technologies that challenge traditional testing paradigms. As such, ECRs can play a central role in advancing the transition toward more ethical, efficient, and human-relevant approaches to chemical risk assessment.

At the same time, the contribution of ECRs is frequently constrained by structural barriers inherent to early-career research, including job insecurity, fragmented professional networks, limited access to mentoring, and uneven opportunities for visibility, training, and leadership (Nature editorial, 2016). Structured support networks can help address these barriers by creating opportunities for scientific exchange, skill development, mentoring, collaboration, and career progression. In rapidly evolving fields such as NAM-based risk assessment, such networks may also accelerate knowledge transfer, strengthen interdisciplinary communities, and support the long-term implementation of new scientific approaches (Valverde et al., 2025; McCarthy et al., 2025). Despite growing recognition of the importance of ECR engagement, practical guidance on establishing, organizing, and sustaining ECR networks within large, multidisciplinary research consortia remains limited. This represents an important gap, particularly in project-based environments where funding is time-limited, coordination is distributed across institutions, and sustained community-building does not arise automatically. A more well-defined framework for designing and maturing ECR networks could therefore be of broad value to NAM-oriented consortia and to other collaborative research settings facing similar challenges.

**Fig. 1:**
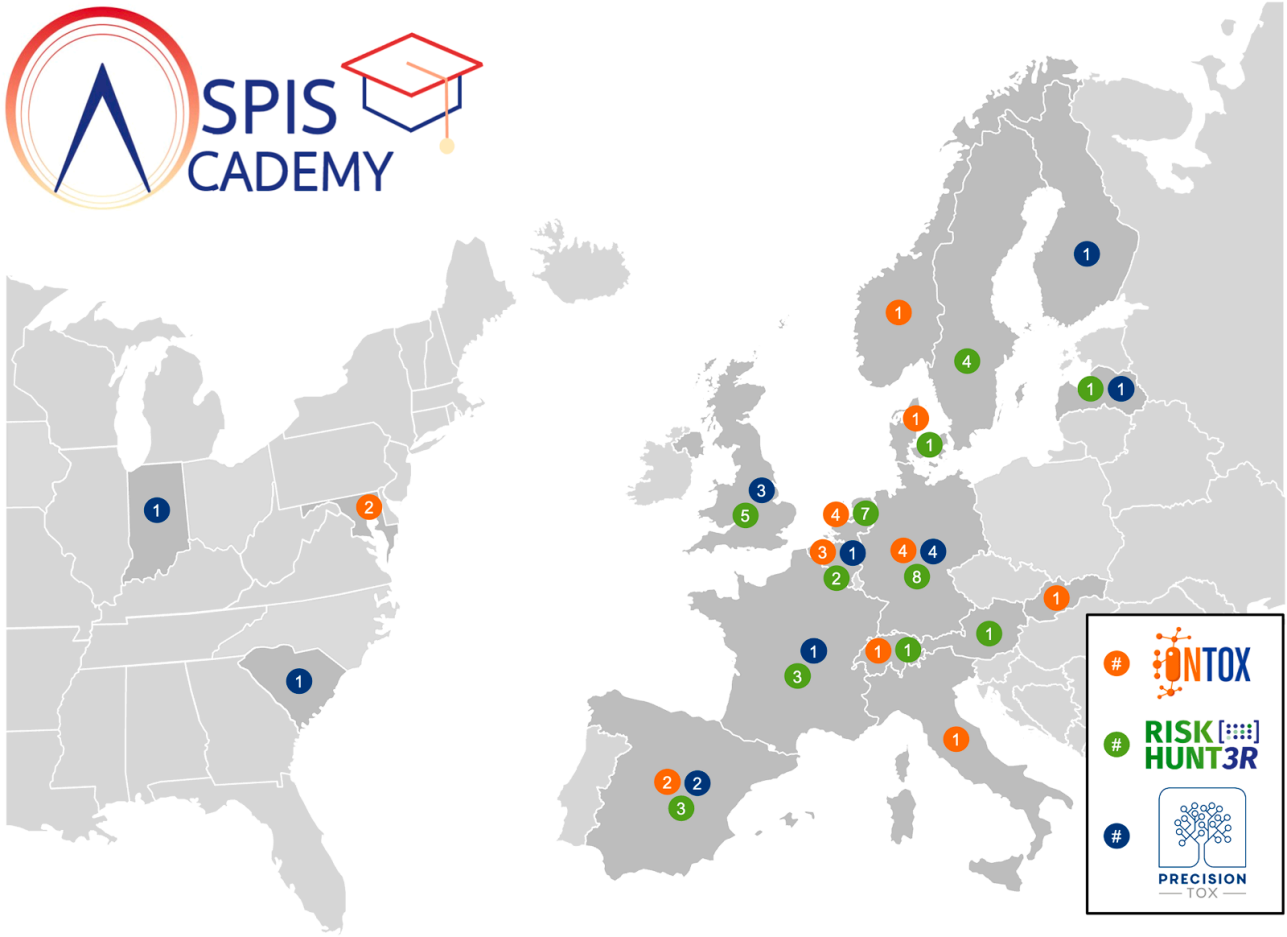
Overview of the ASPIS partners and their geographic representation. The partner institutions of the three ASPIS consortia (ONTOX, RISK-HUNT3R, and PrecisionTox) are shown as the number of partners per country. The figure highlights both past and current ASPIS partners.

The ASPIS cluster provides a particularly relevant setting for bridging this identified gap. Established in 2021, ASPIS stands for “Animal-free Safety assessment of chemicals: Project cluster for Implementation of novel Strategies” and is an international effort, gathering >70 scientific organizations across 16 countries of the European Union, the United Kingdom, and the United States (Fig. 1). ASPIS brings together the EU Horizon 2020-funded projects ONTOX, PrecisionTox, and RISK-HUNT3R in a joint effort to advance animal-free chemical safety assessment through NAM-based approaches (Colbourne et al., 2025; Vinken et al., 2021; The Precision Toxicology initiative, 2023; Pallocca et al., 2022). Within this large and interdisciplinary environment, the ASPIS Academy (AA) was established as a dedicated ECR network to support (primarily) ASPIS early-career scientists through tailored training, mentoring, career development, networking, communication, and collaboration. Beyond supporting individual researchers, AA was conceived as a mechanism to strengthen the ASPIS community's collective capacity to contribute to NGRA.

Here, we use the establishment and evolution of AA as a case study to develop a practical blueprint for building and maturing an ECR network to advance NAM-based chemical risk assessment. Rather than serving solely as a descriptive account of one cluster initiative, this article identifies transferable design principles, governance elements, implementation steps, program components, collaboration strategies, and legacy considerations that may inform the development of similar ECR structures across scientific fields. The sections that follow therefore move from rationale to practice. We first describe how an ECR network can be established and guided toward operational maturity, and then illustrate how such a network can generate scientific, educational, and community-level impact. By presenting both the structure and the lived experience of AA, we aim to show how strategically organized support for ECRs can contribute to the adoption of NAMs while also strengthening the people and relationships that make such transitions possible.

## 2 How to set up an ECR network

Founding an ECR network was a feasible endeavor as several key elements were in place. These include motivated ECRs willing to assume leadership roles, supportive senior advisors (particularly during the initial phase), clear organizational structures, a well-defined goal, flexible time allocation, and, where possible, access to financial resources to support activities and events. This section outlines the key steps required to establish an ECR network and guide it toward operational maturity, illustrated by the case of AA.

**Fig. 2:**
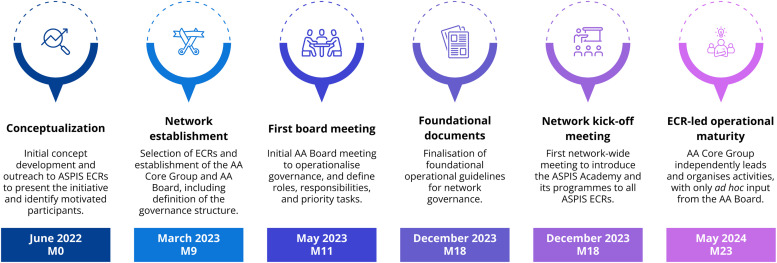
Timeline of early-career network establishment, exemplified by the ASPIS Academy. Abbreviations: ECR, early-career researcher; AA, ASPIS Academy; M, month.

The timeline for establishing the AA network spanned two years, during which full operational maturity was achieved (Fig. 2). Time was a critical factor during the conceptualization phase (hereafter referred to as month 0), especially when the network was embedded within time-limited project funding. At this stage, the overarching goal of the network needed to be clearly defined, for example: “to support the training of the next generation of scientists in the transition from animal testing to NAMs”. The primary outcome of this phase was a strategic plan describing the objectives, scope, and functioning of the future network, exemplified by the AA strategy document **(Supplementary File 1)**. In parallel, a survey was circulated among the target ECR community to identify motivated individuals for network coordination roles and to assess interest in specific career development topics.

Notably, the definition of ECRs may vary across networks. The AA ECR definition aims to be inclusive by defining ECRs as "*individuals who obtained their terminal education diploma within the past decade, encompassing individuals engaged in research sectors, as well as students pursuing master's and bachelor's degrees.*" Additionally, AA embraces inclusivity and welcomes ECRs from diverse backgrounds, beliefs, and identities, fostering equal opportunities for the ideas and dreams of a new generation of scientists to flourish.

In the case of AA, the network was formally established nine months after the conceptualization phase, in March 2023. This timeframe may be shortened depending on context and available resources. Based on survey **(Supplementary File 1)**, distributed to approximately 130 ECRs, two ECRs per ASPIS consortium (six representatives in total) were selected to coordinate the network (referred to as the AA Core Group). The size of the coordination team should be proportional to the network’s size and planned activities. Identifying an appropriate number of ECR coordinators is essential to maintain momentum, particularly in the absence of dedicated funding for coordination and organizational tasks, as was the case for AA. At the inception of AA, all six Core Group members were formally appointed as co-chairs with equal responsibility. In practice, levels of engagement varied over time due to each one’s research projects and obligations, and leadership naturally consolidated around one to two highly committed individuals who assumed primary responsibility for coordination and decision-making. There were no types of financial incentives for volunteering for the network coordination, but the support offered by the leadership of each consortium and the AA Advisory Board. Based on this experience, we recommend that future ECR networks designate a single chair from the outset, ideally supported by a co-chair or chair-elect, to ensure clear leadership, accountability, and continuity. For networks with a longer intended lifespan, it is advisable to rotate ECR coordination members periodically (e.g., every 1 to 4 years). Such rotation helps sustain motivation, encourages the influx of new ideas, and provides leadership experience to a broader group of ECRs. In addition to the AA Core Group, the involvement of senior advisors is strongly recommended during the initial one to two years of the network. Within AA, one senior advisor per consortium, together with the three consortium managers, supported the ECRs in the AA Core Group. Collectively, the six senior advisors and six ECRs formed the AA Board.

Shortly after formal establishment, the first board meeting was held to operationalize governance, define roles and responsibilities, and identify priority tasks. These tasks typically include the organization of initial activities and, critically, the development of foundational operational documents. For AA, these documents were drafted by the AA Core Group, approved by the AA Board, and finalized in December 2023, approximately six months after the first Board meeting. The operational guidelines encompassed the network’s objectives and mission, governance structure (including composition, terms of office, duties, meeting frequency, and reporting), communication strategy, and a description of the network’s programs. The AA operational guidelines (version 2.0, March 2024) are provided in **Supplementary File 2** as a template for other networks, with sensitive information redacted.

In parallel with preparing the operational guidelines, materials for an online network kick-off meeting were developed. The purpose of this meeting was to introduce the network, its programs, and available career development opportunities to the broader ECR community. During the AA kick-off, several supporting tools were launched, including the network webpage (available at https://aspis-cluster.eu/aspis-academy/), a professional networking group on LinkedIn (available at https://www.linkedin.com/groups/9370153/), an anonymous contact mechanism, and a concise starter package summarizing key activities and resources (**Supplementary File 3**, sensitive information redacted). In addition, a structured overview of participating ECRs (including contact details, project/institution involvement, and research interests) was compiled to facilitate networking and collaboration across the ASPIS consortia.

Following the kick-off phase, the ECR Core Group continued to organize activities as described in the subsequent section on programs, initially with regular input from senior advisors. Over time, the ECR Core Group gained experience in leadership, event organization, and strategic planning, gradually reducing its reliance on senior oversight. In AA, this transition culminated in full ECR-led operational maturity in May 2024, approximately 23 months after the initial conceptualization phase. Following the achievement of ECR-led operational maturity, AA progressively expanded its scope and opened selected activities, such as webinars and training events, to external ECRs beyond the ASPIS consortia. Regular monthly meetings of the AA Core Group maintained AA operations until the end of each project.

Finally, regular documentation of activities was an important component of network governance. Annual activity reports provide transparency and continuity and are illustrated by the AA examples in **Supplementary File 4**. Where financial resources are available, internal budget reporting is recommended; however, the AA experience demonstrates that networks can remain viable even without dedicated funding. Substantial contributions can be made on an in-kind basis by ECRs, senior scientists, and invited speakers, particularly within established consortia. Additional cost-efficiency can be achieved by aligning in-person meetings with existing conferences or consortium events and, where appropriate, through limited financial or in-kind support from industrial partners or small and medium-sized enterprises (SMEs). In practice, the financial requirements for core activities were modest. For example, printing and promotional materials amounted to approximately €1000 over the duration of the ASPIS Academy, while catering costs for events such as the Summer School were limited (€200–300). Venue costs were typically avoided by hosting events within partner institutions, and additional support was occasionally provided through partner project budgets.

## 3 ASPIS Academy programs

AA offered a wide variety of programs, which are described in detail in the following sections. The activities organized in these programs are comprehensively further listed in Table 1.

**Table 1:** Overview of ASPIS Academy activities organized from its establishment (2023) until the end of the ASPIS projects (2026). Activities are presented in chronological order; those scheduled after submission of this manuscript (March 2026) are indicated and listed at the end of the table. Where available, recordings or related materials have been made accessible to a broader audience via online platforms (e.g. YouTube), with corresponding links provided in the final column. Abbreviations: AA, ASPIS Academy; NGO, non-governmental organization; NAM, new approach methodology; ECR, early-career researcher; GIVIMP, Good In Vitro Method Practices.

Type	Name	Date	Place	Description/ Aim of the event	Link to the recording or news article of the event
Event	AA kick-off meeting	20.12.2023	Online	Introduce AA programs to members.	https://www.youtube.com/watch?v=yPBgqxbJJTg
Mentoring	1st Career development session	25.01.2024	Online	Introduce different career paths (academia, industry, NGO, intergovernmental organization).	Not available
Training	Connecting with your audience	07.03.2024	Online	How to deliver a dynamic, engaging presentation.	https://aspis-cluster.eu/aspis-academy-career-development/
Conference session	ESTIV congress	03.06.2024–06.06.2024	Prague, Czech Republic	Scientific session as a platform for AA members to present their NAM research and to present the network.	https://aspis-cluster.eu/aspis-academy-championing-estiv/
Training	Finding European research funding and writing a competitive research proposal	21.06.2024	Online	How to find and get research funding.	https://www.youtube.com/watch?v=deLUgzmLDRg&list=PLP7a7oegxbWs-00C5kBi8ACjf-F2zEhbn&index=8
Interview	ASPIS Academy Interview: Mathieu Vinken	01.08.2024	Online	Interview with one of the guests of the 1st career development session.	https://aspis-cluster.eu/aspisaspis-academy-interview-mathieu-vinken/
Interview	ASPIS Academy Interview: Patience Browne	01.08.2024	Online	Interview with one of the guests of the 1st career development session.	https://aspis-cluster.eu/aspis-academy-interview-patience-browne/
Interview	ASPIS Academy Interview: Dylan Underhill	08.08.2024	Online	Interview with one of the guests of the 1st career development session.	https://aspis-cluster.eu/aspis-academy-interview-dylan-underhill/
Interview	ASPIS Academy Interview: Gladys Ouedraogo	15.08.2024	Online	Interview with one of the guests of the 1st career development session.	https://aspis-cluster.eu/aspis-academy-interview-gladys-ouedraogo/
Congress session	ASPIS Open Symposium 2024	11.09.2024	Copenhagen, Denmark	Multiple events to highlight ECRs: oral session, poster session, wine reception, and networking event.	https://aspis-cluster.eu/aspis-academy-aspis-open-symposium-2024/
Video	ASPIS working group series – ASPIS Academy video	12.09.2024	Online	Video about the ASPIS Academy filmed at the ASPIS Open Symposium in Copenhagen.	https://youtu.be/aQ6iu44kQHY?si=RchOj7zgbJvmnCbA
Training	How to Transform Your Research into a Spin-Off Business	12.09.2024	Copenhagen, Denmark	Three inspiring speakers who shared their experiences in launching successful businesses based on scientific research.	https://aspis-cluster.eu/aspis-academy-aspis-open-symposium-2024/
Training	The Importance of Science for Policy and Science Communication for 21st Century Toxicology	17.10.2024	Online	How science shapes policy and why effective communication is key in toxicology.	https://www.youtube.com/watch?v=0JP9TfGWD3c&list=PLP7a7oegxbWs-00C5kBi8ACjf-F2zEhbn&index=9
Video	Introductory video	18.11.2024	Online	Spark interest in AA and its LinkedIn group, directed to a broad audience.	https://www.youtube.com/watch?v=_hLk93NcG9s&list=PLP7a7oegxbWs-00C5kBi8ACjf-F2zEhbn&index=6
Training	2nd ASPIS Academy Career Development session	10.12.2024	Online	Introduce different career paths (consultancy, forensic toxicology, project management, journal publishing).	https://aspis-cluster.eu/aspis-academy-webinar-the-importance-of-science-for-policy-and-science-communication-for-21st-century-toxicology/
Interview	ASPIS Academy Interview: Luisa Orsini	30.01.2025	Online	Interview with one of the speakers of the training on Spin-Off Business.	https://aspis-cluster.eu/aspis-academy-interview-luisa-orsini/
Interview	ASPIS Academy Interview: Kristina Bartmann	30.01.2025	Online	Interview with one of the speakers of the training on Spin-Off Business.	https://aspis-cluster.eu/aspis-academy-interview-kristina-bartmann/
Interview	ASPIS Academy Interview: Leonie Mueller	31.01.2025	Online	Interview with one of the speakers of the training on Spin-Off Business.	https://aspis-cluster.eu/aspis-academy-interview-leonie-mueller/
Training; Collaboration	VHP4Safety/ONTOX webinar 1: Research Output Management Strategies for Animal-Free Risk Assessment	14.02.2025	Online	Disseminate ECRs’ NAM science and showcase the work of the research output management collaboration group between the ONTOX and VHP4Safety projects.	https://www.youtube.com/watch?v=GJ_sU_o-fVM&list=PLP7a7oegxbWumxTHIbfK4qOcZ7RLfEp8M&index=5
Communication	Back-to-School Program lecture	18.02.2025	Brno, Czech Republic	Inform university students about NAMs in developmental neurotoxicity	https://aspis-cluster.eu/aspis-academy-back-to-school/ https://youtu.be/gn_5y-JP92M?si=V_NsyHtNYq5s5VnL
Training; Collaboration	VHP4Safety/ONTOX webinar 2: Bridging Science and Society: Stakeholder Involvement in Animal-Free Risk Assessment	08.04.2025	Online	Disseminate ECRs’ NAM science and showcase the work of the stakeholders engagement collaboration group between the ONTOX and VHP4Safety projects.	https://www.youtube.com/watch?v=kzbmZwAk3MQ&list=PLP7a7oegxbWumxTHIbfK4qOcZ7RLfEp8M&index=4
Training	ASPIS Academy Summer School: “Let’s Talk About Data!”	24.04.2025 - 25.04.2025	Valencia, Spain	A full-day course with interactive sessions led by experts in scientific communication and data management, including a networking evening event.	https://aspis-cluster.eu/aspis-academy-summer-school-2025/ Vlog: https://www.youtube.com/watch?v=pA0VqQ1vYPU
Congress session	ASPIS Academy at SETAC: Bridging the gaps between ESRs for collaborative environmental toxicology communities	11.05.2025 - 15.05.2025	Vienna, Austria	ASPIS Academy, PARC Junior Community, and SETAC Student Advisory Committee collaborative session to present the networks and showcase ECR science.	https://aspis-cluster.eu/aspis-academy-at-setac/
Networking; Collaboration	Kahoot quiz game	27.05.2025	Online	Collaborative quiz game between AA and PARC Junior Community about NAMs and the two projects (clusters).	Not available
Training	VHP4Safety/ONTOX webinar 3: (q)AOPs: advancing human-relevant toxicology through data-driven models.	27.06.2025	Online	Disseminate ECRs’ NAM science and showcase the work of the (q)AOP collaboration group between the ONTOX and VHP4Safety projects.	https://www.youtube.com/watch?v=prFCRESkMXc&list=PLP7a7oegxbWumxTHIbfK4qOcZ7RLfEp8M&index=3
Congress session; Collaboration	World Congress on Alternatives and Animal Use in the Life Sciences (WC13)	02.09.2025	Rio de Janeiro, Brazil	Joint AA, EUROTOX Early Career Forum, ESTIV Early Career Network, Young TPI, and EURL ECVAM Summer School scientific session.	https://aspis-cluster.eu/next-gen-researchers-wc13–2025/
Congress session	ASPIS Open Symposium 2025	17.09.2025–18.09.2025	Athens, Greece	Multiple events to highlight ECRs: oral session, poster session, wine reception, and networking event.	https://aspis-cluster.eu/open-symposium-2025/
Training; Collaboration	VHP4Safety/ONTOX webinar 4: Exploring the ONTOX and VHP4Safety IT platforms.	08.10.2025	Online	Disseminate ECRs’ NAM science and showcase the work of the IT platform collaboration group between the ONTOX and VHP4Safety projects.	https://www.youtube.com/watch?v=RuFqxixCDIY&list=PLP7a7oegxbWumxTHIbfK4qOcZ7RLfEp8M&index=2
Training; Collaboration	VHP4Safety/ONTOX webinar 5: Strategies for next-generation risk assessment in ONTOX and VHP4Safety	18.12.2025	Online	Disseminate ECRs’ NAM science and showcase the work of the risk assessment collaboration group between the ONTOX and VHP4Safety projects.	https://www.youtube.com/watch?v=wERoZAzsq8Y&list=PLP7a7oegxbWumxTHIbfK4qOcZ7RLfEp8M&index=1
Networking	NAM photo competition	13.03.2026	Online	Competition to showcase ECR NAM work more creatively and engagingly.	https://aspis-cluster.eu/aspis-academy_photo-competition/
Training	Hidden Innovators in Toxicology: The untold stories that Shaped Chemical Safety	03.06.2026	Online	Webinar for researchers working in experimental and computational toxicology, NAMs, and chemical safety.	Not available
Congress session; Collaboration	ESTIV Congress 2026	29.06.2026–02.07.2026	Maastricht, the Netherlands	Collaborative scientific session to present the AA network and invited networks (PARC Junior Community, VICT3R ECRs), as well as selection of ECR scientific work to be presented in the Short Orals sessions.	Upcoming event.
Congress session	ASPIS Open Symposium 2026	02.07.2026–03.07.2026	Maastricht, the Netherlands	Multiple events to highlight ECRs: oral session, poster session, wine reception, and networking event.	Upcoming event. https://aspis-cluster.eu/aspis-open-symposium-2026-registration-open/
Training	GIVIMP AA training at the ASPIS Open Symposium	03.07.2026	Maastricht, the Netherlands	In-person training on GIVIMP.	Upcoming event. https://aspis-cluster.eu/open-symposium-givimp-session/

### 3.1 Training program

The AA Training Program aimed to prepare ECRs for their future scientific careers by providing targeted training in various areas. Experienced professionals guided and trained participants in hard skills relevant to NAMs as well as transversal skills necessary for succeess in the field. The activities were therefore specifically tailored to the needs of ECRs and organized by the three member projects, with support from external collaborators as required by the expertise involved. The organization of on-site training activities depended on opportunities to host these events as satellite sessions alongside consortium meetings or related scientific events. The AA Training Program included a varied range of webinars and on-site activities. Among transversal skills, training on science communication, networking, publication and grant writing, the importance of science for policy and science communication for 21st-century toxicology (online, 2024), and the journey from scientific projects to start-ups (Copenhagen, 2024) were held. In parallel, technical skills focused on the application of artificial intelligence in risk assessment (Sitges, 2022), finding European research funding and writing a competitive research proposal (online, 2024), and the ASPIS Academy Summer School. The ASPIS Academy Summer School 2025, held in Valencia under the theme “Let’s Talk About Data!”, represented the most comprehensive training event organized by AA to date. It provided expert-led sessions on research output management, data visualization, and effective communication of NAM-related science, equipping ECRs with key skills for data interpretation and dissemination. The program was complemented by an organized networking evening, including a NAM-related quiz, fostering informal exchange and strengthening community building among participants.

In addition, the AA organized a series of five scientific webinars in collaboration with the Virtual Human Platform for Safety Assessment (VHP4Safety) project (Kienhuis et al., 2024). These webinars (Table 1) were designed to reach a broad audience beyond the ASPIS and VHP4Safety consortia and were open to participants at different career stages. The program covered key topics relevant to NAM-based risk assessment, including research output management, stakeholder involvement, quantitative adverse outcome pathways (qAOPs), IT platforms, and NGRA. In parallel with training objectives, the webinars provided ECRs with dedicated opportunities to present and discuss their research in an open, interdisciplinary setting.

The structure of the program was flexible, with the AA Core Group maximizing opportunities through proactive identification of new training activities and an active role in collecting ECRs' needs to meet their demands. Such a broad program allows ECRs to gain targeted preparation for their current and future careers while dynamically expanding their professional networks and visibility. Most of the online events and initiatives were made available in 2024 and remain publicly accessible (Table 1). Finally, while not directly implemented during the AA’s active phase, mental health emerged as a critical topic repeatedly identified by ECRs. Based on this experience, we recommend that future ECR networks address mental health and well-being explicitly, ideally through dedicated workshops organized early in project lifecycles and repeated as needed in longer-term networks. Proactive engagement with this topic can support sustainable research careers and help mitigate pressures and stress commonly faced by ECRs (Satinsky et al., 2021; Nicholls et al., 2022).

### 3.2 Mirroring program

The AA Mirroring Program aligned ECRs with thematic working groups (WGs) within the ASPIS framework (**Supplementary File 4**), based on individual scientific interests and expertise. Through participation in WGs spanning key methodological and regulatory domains, ECRs engaged in cross-consortia collaboration and gained exposure to expertise beyond their immediate research environment. By embedding ECRs in groups organized around shared scientific interests across the ASPIS consortia, the program supported professional networking and facilitated active scientific contribution. In addition to knowledge exchange, WG participation offered ECRs opportunities to contribute to joint outputs, including active collaboration on scientific publications (Bwanya et al., 2025; Verheijen et al., 2023), thereby complementing their doctoral or postdoctoral research activities. The scientific outcomes and activities of the ASPIS working groups were documented in the publicly available ASPIS annual reports (https://aspis-cluster.eu/annual-reports/).

### 3.3 Twinning program

The AA Twinning Program was designed to support ECRs in acquiring complementary research skills through short-term exchanges with experienced academic or industrial hosts across the ASPIS consortia. It served as a research group exchange program, facilitating connections between ECRs and host institutions based on shared scientific interests and methodological needs. To support this, AA developed the “Manual for ECR mobility from the AA Twinning program” (**Supplementary File 5**), which provided practical guidance on identifying suitable host institutions, defining objectives of a secondment, establishing contact with hosts, and organizing research visits. A key component of the manual was its comprehensive overview of national and European funding opportunities to support ECR-led mobility. The exchange duration was flexible, determined by mutual agreement between hosts and trainees, with financial responsibility resting with the respective institutions. Beyond its role within ASPIS, the Twinning Program Manual was designed as a transferable resource, intended to support ECR mobility and cross-institutional collaboration in future research consortia. Through this initiative, ECRs could gain knowledge, skills, and experience under expert guidance, broaden their exposure to diverse work environments, and nurture and facilitate potential scientific collaborations among the three consortia.

### 3.4 Mentoring program

The AA Mentoring Program provided a structured framework to support ECRs through career development sessions and targeted mentor-mentee interactions. The program focused on career development, knowledge exchange, and professional networking, with mentoring activities tailored to individual needs. Mentors and mentees were matched based on shared scientific and career interests, enabling personalized guidance that supports career progression in the context of NAMs. A total of 15 mentees enrolled in the mentoring program; nearly half were PhD candidates (47%), followed by postdoctoral researchers (33%), with the remaining 20% comprising research fellows, research scientists, and scientific officers. Mentees were primarily affiliated with RISK-HUNT3R (50%), followed by ONTOX (25%) and PrecisionTox (25%). Career interests spanned academia, industry, and regulatory bodies. Most mentees expressed a strong preference for one-to-one online mentoring, as well as interest in group sessions and webinars. They commonly sought career guidance and technical expertise in areas such as physiologically based pharmacokinetic (PBPK) modelling, quantitative structure-activity relationship (QSAR) modelling, and AOP modelling, as well as support in developing transversal skills, including communication, leadership, and work-life balance skills. On the other hand, 26 mentors registered for the Mentoring Program. This resulted in not all mentors being matched to a mentee, although it highlighted the eagerness of senior scientists in supporting AA activities and ECRs. While the mentor cohort consisted predominantly of senior scientists and principal investigators, it also included postdoctoral researchers mentoring PhD students, thereby gaining structured mentoring experience themselves. Half of the mentors were affiliated with ONTOX, followed by RISK-HUNT3R (31%) and PrecisionTox (19%). The mentor cohort demonstrated extensive expertise across academia, industry, regulatory science, and policy, with strengths in NAMs, and in vitro and *in silico* toxicology, including PBPK, QSAR, toxicokinetic modelling, toxicogenomics, and artificial intelligence. Feedback was collected from participating mentees, all of whom confirmed meeting with their assigned mentor at least once during the mentoring cycle. Most respondents (80%) reported that their interactions occurred via virtual meetings, while 20% reported meeting in person. Overall, the feedback was highly positive, with all mentees expressing satisfaction with the mentoring experience and indicating that their mentors met their expectations, describing the meetings as useful and aligned with their needs.

Two career development sessions were designed to provide ECRs with structured insights into diverse career paths relevant to NAMs. The first session, held in January 2024, brought together speakers representing key career trajectories, including industry (Gladys Ouedraogo, L’Oréal), intergovernmental organization (Patience Browne, OECD), non-governmental organization (NGO, Dylan Underhill, Cruelty Free International), and academia (Mathieu Vinken, Vrije Universiteit Brussel). Speakers shared perspectives on their professional roles, daily work environments, and career transitions, with a particular focus on the transition from academia to industry, regulation, and NGOs, as well as differences in work culture and responsibilities across sectors. The second career development session took place in December 2024 and focused on additional career options frequently less visible to ECRs. Speakers represented scientific publishing (Jagna Gent-Aalén, Elsevier), project management (Martijn Moné, RISK-HUNT3R), forensic toxicology (Daniel Dietrich, University of Konstanz), and scientific consulting (Paul Whaley, Whaley Research). Each speaker outlined their career trajectory, key milestones, and the transferability of skills across sectors, followed by an interactive Q&A session. >50 ECRs from all three ASPIS consortia participated in these sessions, enabling a lively discussion. Additionally, ECR-led interviews were conducted with guests of the first session.

### 3.5 Communication and dissemination

Communication and dissemination (C&D) are integral to building a visible, connected, and effective scientific community. The AA’s C&D strategy focused on purpose-driven outreach to promote educational activities, scientific outputs, and networking opportunities, with a primary emphasis on ECRs and on the broader scientific community. AA was advised on C&D topics by the ASPIS C&D working group, which also helped increase the outreach. There are several channels that were established for C&D purposes, both internally (toward the parent ASPIS cluster) and externally, reaching a broader scientific audience and ECR networks. The central communication hub of AA was its dedicated section within the ASPIS cluster website (https://aspis-cluster.eu/aspis-academy/), which provided up-to-date information on AA’s scope, activities, Core Group, outputs, calendar of upcoming and past events, and contact points. This platform is complemented by additional C&D channels, including social media, a YouTube channel (https://www.youtube.com/@ASPIScluster), newsletters, conference sessions, and targeted networking events, enabling tailored dissemination to different audiences. Social media dissemination was primarily conducted through the channels of ASPIS cluster partners, supplemented by the AA LinkedIn Group (opened to ECRs outside of ASPIS in 2024). Consistent branding (such as stickers distributed at ASPIS booths at conferences), strategic use of visuals and hashtags, and cross-platform promotion supported recognizability and engagement. YouTube served as a key medium for sharing recorded webinars and training sessions, facilitating access to educational content and supporting the communication of complex NAM-related topics.

In addition, AA conducted interviews with researchers, trainers, and partners to highlight expertise and career pathways. These interviews were published as news items on the AA webpage, and disseminated via social media, strengthening community cohesion and providing accessible entry points into specialized scientific areas. Importantly, the process of designing, conducting, and moderating these interviews also served as a training opportunity for ASPIS ECRs. This enabled them to develop science communication, interviewing, and outreach skills, thereby creating reciprocal benefits for both interviewees and interviewers.

AA actively engaged in conferences as a central element of its C&D strategy, using these venues to both showcase ECR-led science and strengthen inter-network collaboration (Table 1). Three complementary types of conference sessions were organized. The first type focused on providing dedicated platforms for ECRs from ASPIS and partner networks to present their scientific work, thereby increasing visibility of ECR-led NAM research and supporting presentation experience at international meetings. These sessions were implemented, for example, at European Society of Toxicology In Vitro (ESTIV) congress 2024 (AA ECRs only), Society of Environmental Toxicology and Chemistry (SETAC) congress 2025 (AA, Partnership for the Assessment of Risks from Chemicals (PARC) Junior Community, and SETAC Student Advisory Committee), and the upcoming ESTIV congress 2026 (AA, PARC Junior Community, and VICT3R ECRs). The second type comprised sessions explicitly dedicated to ECR networks, their establishment, and collaboration building, highlighting the role of structured networking initiatives in advancing NAM uptake. A notable example was the session organized at World Congress 13 (Rio de Janeiro, 2025), which brought together AA alongside EUROTOX Early Career Forum, ESTIV Early Career Network, Young TPI, and the EU Joint Research Center (JRC) Summer School to showcase complementary network activities and demonstrate the added value of inter-network collaboration. The third type was linked to a parent conference – the annual ASPIS Open Symposium, where AA co-organized a dedicated poster session for ECRs with a best-poster competition and networking. In addition to organized sessions, AA Core Group members actively disseminated information during scientific meetings and congresses through posters and presentations, strengthening network visibility while providing ECRs with experience in scientific outreach and advocacy.

Additionally, AA organized dedicated educational and outreach initiatives. The Back-to-School Program focused on motivating ECRs to reconnect with their former high schools or universities to present their NAM-related research (Table 1). This initiative fostered early awareness of human-relevant toxicology approaches among students while encouraging ECRs to actively engage in science communication and outreach beyond the academic community. Finally, lessons learned from establishing the network were also disseminated beyond ASPIS, for example, through a dedicated workshop on the management of EU Horizon projects organized by the ONTOX consortium at the ESTIV Congress in 2024. Finally, AA reported on its achievements in the annual ASPIS cluster reports (**Supplementary File 4**) and European Centre for the Validation of Alternative Methods (ECVAM) reports (Zuang et al., 2025; Barroso et al., 2024).

## 4 Collaborations

Collaboration between ECR networks is a key driver for accelerating the development, acceptance, and implementation of NAMs. By sharing expertise and creating joint platforms for exchange and visibility, inter-network collaboration amplifies scientific impact and supports sustainable community building. Accordingly, collaboration with other ECR initiatives was a core strategic priority of AA. These collaborations can be formally strengthened by establishing a Memorandum of Understanding, as in the case of AA and the European Society of Toxicology In Vitro (ESTIV), VHP4Safety (Kienhuis et al., 2024), and the PARC project (Marx-Stoelting et al., 2023).

AA organized scientific sessions at international conferences in collaboration with established ECR networks (see sections above and Table 1), providing platforms to present network structures, share experiences in establishing and growing networks, and showcase scientific work by ECR members. These activities were carried out together with the PARC Junior Community, the SETAC Student Advisory Council, the EUROTOX Early Career Forum, young TPI (Valverde et al., 2025), and the ESTIV Early Career Network (Abdallah et al., 2026), and contributed to cross-network knowledge transfer and visibility. Joint webinars were also organized to highlight NAM-related research conducted by ECRs and to facilitate interaction between different communities. This format provided ECRs with dedicated presentation opportunities while fostering exchange across initiatives, as exemplified by collaboration with the VHP4Safety project. In addition, AA actively supported the establishment and development of emerging ECR networks by sharing practical experience and providing targeted advice, including through dedicated exchanges with the VICT3R ECR group of the VICT3R project (Sanz et al., 2025). Finally, collaborative networking activities, both online and in person, were organized to strengthen professional connections among ECRs, including informal joint events with the PARC Junior Community, and EUROTOX conference-linked networking activities co-organized with the EUROTOX Early Career Forum.

## 5 Evidence of impact and limitations

The ASPIS Academy was not designed as a formal intervention study, and no prospective framework was established to evaluate its impact. For this reason, the evidence presented here should be interpreted as descriptive rather than causal. Nevertheless, several observations suggest that AA provided meaningful support to ECRs within a large, distributed consortium. It helped organize an active community of approximately 130 ASPIS ECRs into a more visible and coordinated network, with defined roles, regular activities, and shared programs.

In practice, one of the clearest signs of AA’s value was that ECRs and senior colleagues continued to ask for, organize, attend, and contribute to new activities. The Mentoring Program illustrates both the promise and the complexity of this type of initiative. 15 mentees enrolled, while 26 mentors registered, so not all mentors could be matched. Rather than treating this only as a numerical imbalance, we interpret it as evidence of strong willingness among senior scientists to support ECR development. At the same time, because feedback was collected only from enrolled participants, we cannot determine why other ECRs chose not to register or whether the format met the needs of the wider community. Future networks would benefit from collecting feedback from both participants and non-participants from the outset. Feedback from participating mentees was positive, with respondents reporting that the mentoring interactions were useful and aligned with their expectations. Career development activities also attracted broad participation, with >50 ECRs attending two sessions on career pathways relevant to NAMs. These sessions were valuable not only because they presented different professional options, but also because they addressed a recurring need among ECRs: understanding what a career in NAM-related science can look like beyond the immediate doctoral or postdoctoral project.

AA also generated educational and community-building outputs beyond the immediate ASPIS consortia. These included a broad training portfolio, the 2025 in-person Summer School attended by 34 ECRs, and five webinars co-organized with VHP4Safety, which together reached 200 live participants and 202 additional YouTube views by 24 March 2026. As the network matured, selected webinars and training activities were opened to external ECRs, extending AA's reach and strengthening exchange with other communities. Joint conference sessions, webinars, and collaborations with other ECR networks further increased visibility and created opportunities for ECRs to present their work, practice communication, and connect across projects.

These observations should be considered alongside important limitations. The evidence is based primarily on participation numbers, program delivery, and participant feedback rather than on pre- and post-assessment, comparator groups, or long-term follow-up. We therefore cannot determine the extent to which AA directly influenced career progression, sustained collaboration, or downstream uptake of NAMs. This limitation reflects the reality in which the network was created: AA was developed and run by volunteer ECRs, whose early energy was directed toward making the network function and responding to immediate community needs rather than evaluating it as a research object. We therefore recommend that future ECR networks incorporate simple, prospective evaluation measures from the outset, such as engagement tracking, feedback from non-participants, and follow-up questions on confidence, sense of community, leadership development, and collaboration.

A further limitation concerns sustainability. AA relied heavily on highly committed individuals, with no dedicated budget for administrative support or protected time for coordination. The network benefited from the support of consortium coordinators, advisors, supervisors, and project teams, but this support cannot be assumed in every setting. If ECR leadership, visibility, and integration into project governance are expected by funders and advisory boards, then future projects should allocate resources for these activities during the planning phase. Volunteer energy can build a network, but it should not be the only structure holding it together.

## 6 Legacy and future perspectives

Shortly after establishing an ECR network, it is advisable to systematically reflect on its long-term sustainability, including strategic perspectives for the next five to ten years and the anticipated legacy it will leave should the network cease to operate. This consideration is particularly critical for networks that are primarily dependent on time-limited project funding, as is the case for AA, given that funding for the ASPIS projects will end in October 2026. In this context, AA has identified three realistic future scenarios. First, the network could continue through new project-based funding, for example, via a potential new NAMs-oriented cluster. However, such opportunities are inherently uncertain and may only become clear later. In addition, with this possibility in place, it is advisable for projects aiming to involve ECRs in leadership and decision-making processes, or to build such networks as the AA, to include these plans in the budget planning for new consortia. In this way, a stable source of funding for developing network activities is secured, enabling the network to succeed. Second, AA could seek alternative funding mechanisms through competitive calls for networking and capacity-building, such as the European Cooperation in Science and Technology (COST) Actions (https://www.cost.eu/). Third, the network could be integrated into or merged with existing ECR networks or societies that benefit from more stable, long-term funding structures such as ESTIV and EUROTOX. While these options are specific to AA, they are broadly applicable to other ECR initiatives. Importantly, networks should assess the advantages and limitations of each pathway well in advance of funding termination, ideally two years beforehand.

**Fig. 3:**
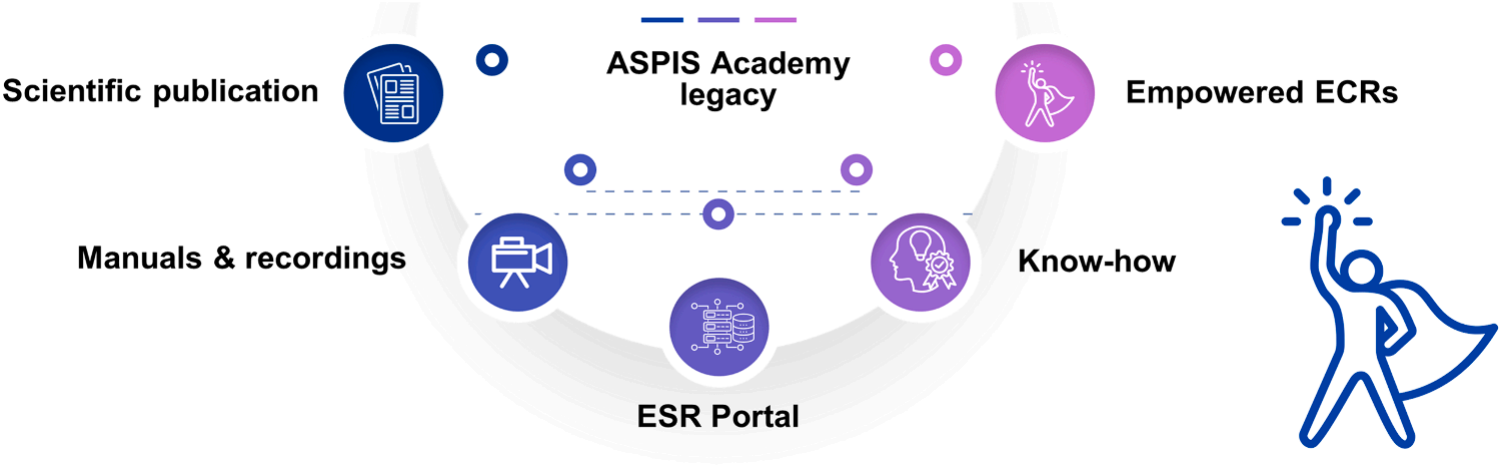
Key legacy elements of the ASPIS Academy. ESR portal refers to the Early-Stage Researcher Assistant (ESRA). Abbreviations: ECR, early-career researcher.

In parallel with sustainability planning, it is essential to define tangible and intangible legacy outcomes that persist beyond the network’s active lifetime. From this perspective, AA has identified several key legacy elements (Fig. 3).

The AA core group accumulated substantial practical experience in establishing, coordinating, and sustaining a functional ECR network tailored to the needs of early-career scientists. This accumulated know-how has been consolidated into the present manuscript, which is explicitly intended to serve as a blueprint for project coordinators, ECR representatives, and other stakeholders aiming to establish comparable networks. Moreover, the network served as a formation space in which it is expected that the ECRs who led the network acquired experience and expertise and will possibly carry this throughout their careers, reproducing and expanding support for new young scientists and guiding the next generations of toxicologists.

Second, AA has generated a substantial body of reusable materials that will remain accessible after the conclusion of ASPIS. These include, for example, the Twinning Program manual (**Supplementary File 5**), which provides structured guidance for planning ECR internships, as well as recorded webinars, training sessions, and meetings. Some of these materials are publicly available on the ASPIS YouTube channel and serve as enduring educational resources on NAMs.

Third, building on practical experience within AA, the concept of an Early-Stage Researcher Assistant (ESRA) was developed by two core group members, Devon Barnes and Rita Ortega-Vallbona. ESRA is envisioned as a pan-European web-based portal designed to support ECRs in navigating the fragmented landscape of training opportunities, events, and networks. Early-career researchers, particularly at the start of their PhD training, often lack an overview of relevant networks and career development opportunities within their field. ESRA aims to serve as a central digital hub where multiple ECR networks can showcase their profiles and advertise activities, thereby reducing informational barriers and facilitating engagement. This concept is under development and its submission to competitive funding calls is in preparation.

Fourth, a major and less tangible legacy lies in the transfer of leadership expertise to other initiatives. Two founding co-chairs of AA, Eliska Kuchovska and Luiz Ladeira, as well as a Core Group member, Peter Pôbiš, have directly applied the knowledge and experience gained within ASPIS to the establishment and strategic development of new ECR networks. Luiz Ladeira and Peter Pôbiš are actively involved in shaping the ESTIV Early Career Network (Abdallah et al., 2026), while Eliska Kuchovska serves as founding chair of the EUROTOX Early Career Forum (https://www.eurotox.com/ecf-about/). Ongoing discussions between these networks aim to reuse and disseminate the AA materials across broader ECR communities, thereby extending their impact.

Fifth, the core legacy of AA resides in the ECRs themselves. Through participation in the AA activities, ECRs have been empowered with scientific, technical, and conceptual knowledge related to NAMs, as well as with transferable skills in career development, networking, communication, and dissemination. Beneficiaries include ASPIS-affiliated ECRs who engaged in targeted activities such as mentoring programs, summer schools with hands-on training, and career development events, as well as non-ASPIS ECRs who accessed public activities, particularly webinars. In addition, students at the university and secondary school levels were reached through the “Back-to-School” Program, in some cases representing their first exposure to NAM-related concepts. Collectively, these individuals constitute a sustained human-capital legacy that extends well beyond the formal duration of AA. As ECRs mature and progress in their careers, no longer being ECRs, it is natural that they will continue to open doors for other ECRs and promote the future of their mentees.

Taken together, these experiences demonstrate that early integration of legacy planning into the design and governance of ECR networks is essential to maximize long-term scientific, educational, and societal impact beyond the constraints of project-based funding.

## 7 Conclusion

The AA demonstrates that an ECR-led network can be established and sustained within a large, multidisciplinary research cluster, even when resources are limited and coordination depends heavily on voluntary commitment. Through training, mentoring, mirroring, twinning, communication, dissemination, and collaboration with other networks, AA created practical opportunities for ECRs to develop scientific, professional, and leadership skills relevant to NAM-based chemical risk assessment. The value of AA lies not only in the activities delivered, but also in the experience gained by the ECRs who built and participated in the network. AA provided a space to test leadership, ask career questions, present work, build collaborations, and learn how to make NAM science visible to different audiences. Its progression toward ECR-led operational maturity shows that early responsibility, when combined with senior support and trust, can strengthen both individual researchers and the wider scientific community.

Beyond ASPIS, AA leaves behind reusable resources, leadership experience, and collaborative practices that can inform future ECR initiatives. More importantly, it leaves behind researchers who have benefited from early support and may carry these practices into the next stages of their careers. Investing in ECR networks is therefore not only a strategy for capacity building; it is a way of shaping the people, relationships, and scientific culture needed to advance more ethical, human-relevant, and sustainable chemical risk assessment.

## List of abbreviations

3Rs: Replacement, reduction, and refinement.

AA: ASPIS Academy.

AOP: Adverse Outcome Pathway.

ASPIS: Animal-free Safety assessment of chemicals: Project cluster for Implementation of novel Strategies.

C&D: Communication and dissemination.

COST: European Cooperation in Science and Technology.

ECR: Early-career researcher.

ECVAM: European Centre for the Validation of Alternative Methods.

ESR: Early-stage researcher.

ESRA: Early-Stage Researcher Assistant.

ESTIV: European Society of Toxicology In Vitro.

EU: European Union.

EUROTOX: Federation of European Toxicologists & European Societies of Toxicology.

GIVIMP: Good In Vitro Method Practices.

JRC: Joint Research Center.

NAM: New approach methodology.

NGO: Non-governmental organization.

NGRA: Next-generation risk assessment.

OECD: Organisation for Economic Co-operation and Development.

PARC: Partnership for the Assessment of Risks from Chemicals.

PBPK: Physiologically based pharmacokinetic. qAOPs: Quantitative adverse outcome pathways.

QSAR: Quantitative structure-activity relationship.

SETAC: Society of Environmental Toxicology and Chemistry.

SMEs: Small and medium-sized enterprises.

VHP4Safety: Virtual Human Platform for Safety Assessment.

WGs: Working groups.

## Use of AI tools

Language editing support was provided using AI-based writing assistants (ChatGPT, Grammarly), which were used to improve clarity, shorten text passages, and refine phrasing; no content or data interpretation was generated by the AI. After using this tool/service, the authors reviewed and edited the content as needed and take full responsibility for the publication’s content.

## Funding

This work was performed as part of the ASPIS cluster (https://aspis-cluster.eu/). The ASPIS cluster has received funding from the European Union’s Horizon 2020 Research and Innovation programme under grant agreement No 963845 (ONTOX - https://ontox-project.eu/), No 965406 (PrecisionTox - https://precisiontox.org/), and No 964537 (RISK-HUNT3R - https://www.risk-hunt3r.eu/).

## CRediT authorship contribution statement

**Luiz Ladeira:** Writing – review & editing, Writing – original draft, Visualization, Investigation, Conceptualization. **Ruben Martinez:** Writing – review & editing, Investigation. **Gaëlle Hayot:** Writing – review & editing, Investigation. **Barira Islam:** Writing – review & editing, Investigation. **Christina H.J. Veltman:** Writing – review & editing, Investigation. **Julen Sanz-Serrano:** Writing – review & editing, Writing – original draft, Supervision, Investigation. **Hiba Khalidi:** Writing – review & editing, Writing – original draft, Investigation. **Rita Ortega-Vallbona:** Writing – review & editing, Writing – original draft, Visualization, Investigation. **Marie Corradi:** Writing – review & editing, Writing – original draft, Visualization, Investigation. **Eike Cöllen:** Writing – review & editing, Writing – original draft, Investigation. **A. Melina Steinbach:** Writing – review & editing, Writing – original draft, Investigation. **Klára Gabrišová:** Writing – review & editing, Writing – original draft, Investigation. **Peter Pôbiš:** Writing – review & editing, Writing – original draft, Investigation. **Devon Barnes:** Writing – review & editing, Investigation. **Shaleen Glasgow:** Writing – review & editing, Investigation. **Marije Niemeijer:** Writing – review & editing, Visualization, Investigation. **Julia Dominika Zajac:** Writing – review & editing, Project administration, Conceptualization. **Agata Ormanin-Lewandowska:** Writing – review & editing, Project administration, Conceptualization. **Martijn J. Moné:** Writing – review & editing, Project administration, Conceptualization. **Bob van de Water:** Writing – review & editing, Funding acquisition, Conceptualization. **John K. Colbourne:** Writing – review & editing, Funding acquisition, Conceptualization. **Mathieu Vinken:** Writing – review & editing, Funding acquisition, Conceptualization. **Silvia Tangianu:** Writing – review & editing, Supervision. **Helena Kandarova:** Writing – review & editing, Supervision. **Giorgia Pallocca:** Writing – review & editing, Supervision, Conceptualization. **François Busquet:** Writing – review & editing, Supervision, Conceptualization. **Eliska Kuchovska:** Writing – review & editing, Writing – original draft, Visualization, Supervision, Project administration, Investigation, Conceptualization.

## Declaration of competing interest

The authors declare the following financial interests/personal relationships which may be considered as potential competing interests:

Given Mathieu Vinken’s role as editor-in-chief of the NAM journal, he had no involvement in the peer review of this article and had no access to information regarding its peer review. Full responsibility for the editorial process for this article was delegated to another journal editor.

Given Helena Kandarova’s role as editor of the NAM journal, she had no involvement in the peer review of this article and had no access to information regarding its peer review. Full responsibility for the editorial process for this article was delegated to another journal editor.

If there are other authors, they declare that they have no known competing financial interests or personal relationships that could have appeared to influence the work reported in this paper.

## Data Availability

No data was used for the research described in the article.

## Appendix Supplementary materials

Image, application 1

Image, application 2

Image, application 3

Image, application 4

Image, application 5
