# Supplementary material for: ASPIS Academy: A blueprint for building early-career researcher networks to advance NAM-based risk assessment
Source: NAM J. 2026 Aug 7;2:100124. doi: 10.1016/j.namjnl.2026.100124 (PMC13482515; doi:10.1016/j.namjnl.2026.100124)
Supplement: Supplementary file 1 [file mmc1.pdf]

ASPIS ACADEMY STRATEGY PLAN

BACKGROUND

- In November 2021, at the ASPIS cluster's kick off symposium in Brussels, interest in forming an ESR (early stage researchers) cluster network was raised by the European Commission participants and coordinators.
- This suggestion was taken on board by the Communication and dissemination Working Group (WG).

STRATEGY

- In June 2022, a survey was distributed to the three consortia addressing the ESRs (see Annex 1 in a separate document).
  The aim was to poll preferences on focus topics for career development sessions and to identify an interest in establishing an ASPIS ESR network, tentatively named ASPIS Academy
- The survey was filled by ~20 ESRs among the three consortia. Possible focus topics for training and platform were suggested by the participants.

Survey results

The replies to question 2 can be found in Figure 1.

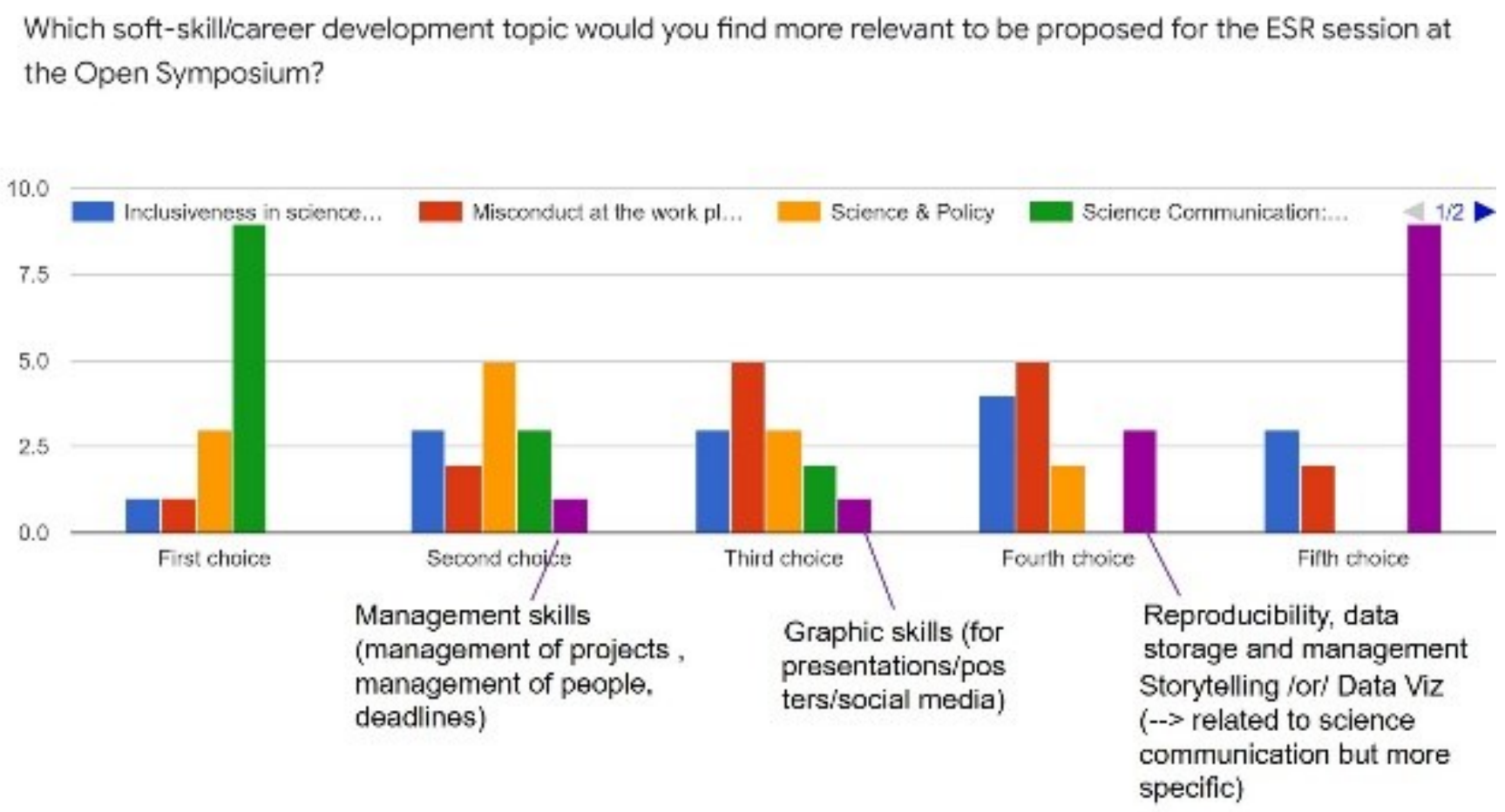

FIGURE 1

The preferences proposed for question 5 are summarized in Table 1.

**Which topics would you find important to share and discuss in such group?**
• sharing interesting events for career development; invite guests talking about their careers/teaching science communication; motivate ourselves to create/create together during the meetings communication/dissemination things useful for our projects (short videos, interviews, ...); share interesting scientific competitions; etc.
• the future of toxicology and how to navigate PhD and postdoc, what to prioritize, how to make it YOUR field
• PBPK modeling, IVIVE, AOP
• Finding your voice as an ESR in a big consortium - or more generally exchange on challenges faced by ESR in ASPIS
• chemical and concentrations selection
• The data generated, in order to be used for other WPs
• personal and project related progress and development
• Topics related to communication (science and self-promotion), funding gathering, academia-industry interface and collaboration, how to lead your own team (for starters), networking advice, good practices in science communication (from scientific papers to newspapers), how to advise students efficiently, and so on!
• Careers in/outside academia, scientific communication, transversal skills

TABLE 1

PURPOSES

- A viable ESR network focused on the use of Novel Approach Methodologies (NAM) for chemical risk assessment can be considered an efficient dissemination tool, allowing, in the long term, to integrate expertise in diverse organizations when entering the job market.
- Over the duration of the cluster, WG on communication and dissemination will intend to foster and support ESR needs and initiatives in order to build and set up a new generation of ESR well educated in NAMs and its functioning ecosystem.

OBJECTIVES

- Build a viable and sustainable framework (website, visual, mailing list, communication channels.);
- Support yearly activities as webinars or face to face meetings;
- Organise poster sessions at GA meetings;
- Impulse social events;
- Coordinate ESR needs.

VISUAL IDENTITY

- A logo has been designed by RISK-HUNT3R (Figure 2).

FIGURE 2

A webpage could be dedicated to this activity on the ASPIS' website based on the content of this document (after approval). Moreover, cluster training activities or webinars should be listed. Public level of this subpage must be further discussed.

FUNCTIONING

- A core group of ESR members (from 1 to 2 representatives per consortium) should be ideally formed to represent a contact point among the ESR network and Comm&Diss WG.
- ESR core members will be regularly invited to the Comm&Diss WG TCs to discuss ESR future activities with the members, to feed the discussion and facilitate communication.
- ESR network members will (virtually) meet, in an early stage with the support of WG Diss&Comm members, in a later stage independently.

CURRENT ACTIVITIES

- The ASPIS Open Symposium at ESTIV congress 2022 hosts two training sessions on science communication and AI for reproducible research as well as a social event and distribution of a poster award. See registration formular.

CHALLENGES

- There is no current budget allocated for this specific activity. Moreover, PIs are limited to send ESRs from their group to extra congresses/events for which travel budget was not previously considered.
- Previous Ph.D. students that were part of EU-ToxRisk consortium were contacted over the summer to participate as alumni to the network. General feedback was positive but limited resources for F2F participation.
- Clear identification of the added value of such a network and training offer must defined, when compared with already available alumni groups (e.g .Marie Curie programs) and online webinars.
- The ASPIS cluster can't be a formal a training program.

ASPIS cluster ESR group - activities at the 2nd ASPIS Open Symposium (Sitges 24-25 Nov 2022)

*Required

1. Email *

____

2. Which soft-skill/career development topic would you find more relevant to be proposed for the ESR session at the Open Symposium? *

Mark only one oval per row.

	Inclusiveness in science (gender, LGBTQIA+, minority groups)	Misconduct at the work place	Science & Policy	Science Communication: how to promote yourself and your research	Other (please specify below)
**First choice**	⬭	⬭	⬭	⬭	⬭
**Second choice**	⬭	⬭	⬭	⬭	⬭
**Third choice**	⬭	⬭	⬭	⬭	⬭
**Fourth choice**	⬭	⬭	⬭	⬭	⬭
**Fifth choice**	⬭	⬭	⬭	⬭	⬭

3. If choose "other", please specify here.

____

Become an active member of the cluster	The creation of a ASPIS Working (sub-) Group coordinated by the ESR of the three consortia has been proposed. We would be interested in collecting preliminary interest in participating in such activity.

4. Would you be interested in participating/coordinating an ASPIS WG for the ES researchers of the cluster? *

Mark only one oval.

- Yes
- No
- Maybe

5. Which topics would you find important to share and discuss in such group?

6. Comments

This content is neither created nor endorsed by Google.

Google Forms
