# Supplementary material for: ASPIS Academy: A blueprint for building early-career researcher networks to advance NAM-based risk assessment
Source: NAM J. 2026 Aug 7;2:100124. doi: 10.1016/j.namjnl.2026.100124 (PMC13482515; doi:10.1016/j.namjnl.2026.100124)
Supplement: Supplementary file 2 [file mmc2.pdf]

ASPIS Academy: A Blueprint for Building Early-Career Researcher Networks to Advance NAM-Based Risk Assessment

SUPPLEMENTAL FILE 2 – AA operational guidelines (version 2.0, March 2024) featuring the founding members of the ASPIS Academy active in 2023, with an updated description of the roles in 2024.

COMMUNICATION PLAN GOVERNANCE PROGRAMS

ASPIS Academy

These projects have received funding from the European Union's Horizon 2020 research and innovation programme under grant agreement No. 963845 (ONTOX), No. 965406 (PrecisionTox). No. 964537 (RISK-HUNT3R)

Contents

1. INTRODUCTION AND OBJECTIVES 3
2. GOVERNANCE DOCUMENT 4
  2.1 Purpose & objectives 4
  2.2 Composition 4
  2.3 Duties 5
  2.4 Meeting frequency 5
  2.5 Reporting requirements 6
  2.6 Management 6
3. COMMUNICATION PLAN 7
  3.1 Communication & dissemination strategy & target audiences 7
    Communication strategy 7
    Target audiences 9
  3.2 Communication & dissemination tools & activities 10
    Project logo 10
    ASPIS Academy Starter Pack 10
    Activities 10
    Potential collaborations with other ECR networks 11
  3.3 Communication & dissemination channels 12
  3.4 Sustainability plan 12
4. PROGRAMS 13
  4.1 ASPIS Academy Mentoring Program 13
  4.2 ASPIS Academy Twinning Program 15
  4.3 ASPIS Academy Mirroring Program 16
  4.4 ASPIS Academy Training Program 17

1. INTRODUCTION AND OBJECTIVES

ASPIS cluster is a collaborative effort of the H2020-funded projects ONTOX, PrecisionTox, RISK-HUNT3R and represents Europe's €60M effort towards the sustainable, animal-free and reliable chemical risk assessment of tomorrow. It stands for "Animal-free Safety assessment of chemicals: Project cluster for Implementation of novel Strategies".

The purpose of the **ASPIS Academy (AA)** is to build a viable early-stage researchers (ECRs) network focused on the use of New Approach Methodologies (NAMs) for chemical risk assessment. The ASPIS Academy promotes the careers of ECRs by providing specialized training, promoting equal opportunity for everyone and by creating a platform devoted to the voices and aspirations of a new generation of young scientists who are the natural experts of NAMs and their applications.

The AA Board is the primary governing body of the AA responsible for supervising and controlling the management and operation of the AA. The members of the AA Board and their duties are outlined in section 3 of this document. 2 ECR representatives of each project form the **AA Core group** and are part of the AA Board.

Early-stage researchers are defined as researchers who received their terminal education diploma not more than 10 years ago. Researchers working both in academia and industry as well as master and bachelor students are included. Exemptions may apply if event capacity and budget allow it.

This document includes the communication strategy, governance, and description of different programs of the AA.

2. GOVERNANCE

2.1 Purpose & objectives

The ASPIS Academy Board is the primary governing body of the ASPIS Academy, responsible for supervising and controlling the management and operation of the Academy and shall report to the ASPIS Coordination Team.

ASPIS Academy MISSION

The purpose of the ASPIS Academy is to build a viable early-stage researchers (ECRs) network focused on the use of New Approach Methodologies (NAMs) for chemical risk assessment. The ASPIS Academy promotes the careers of ECRs by providing specialized training, promoting equal opportunity for everyone and by creating a platform devoted to the voices and aspirations of a new generation of young scientists who are the natural experts of NAMs and their applications.

2.2 Composition

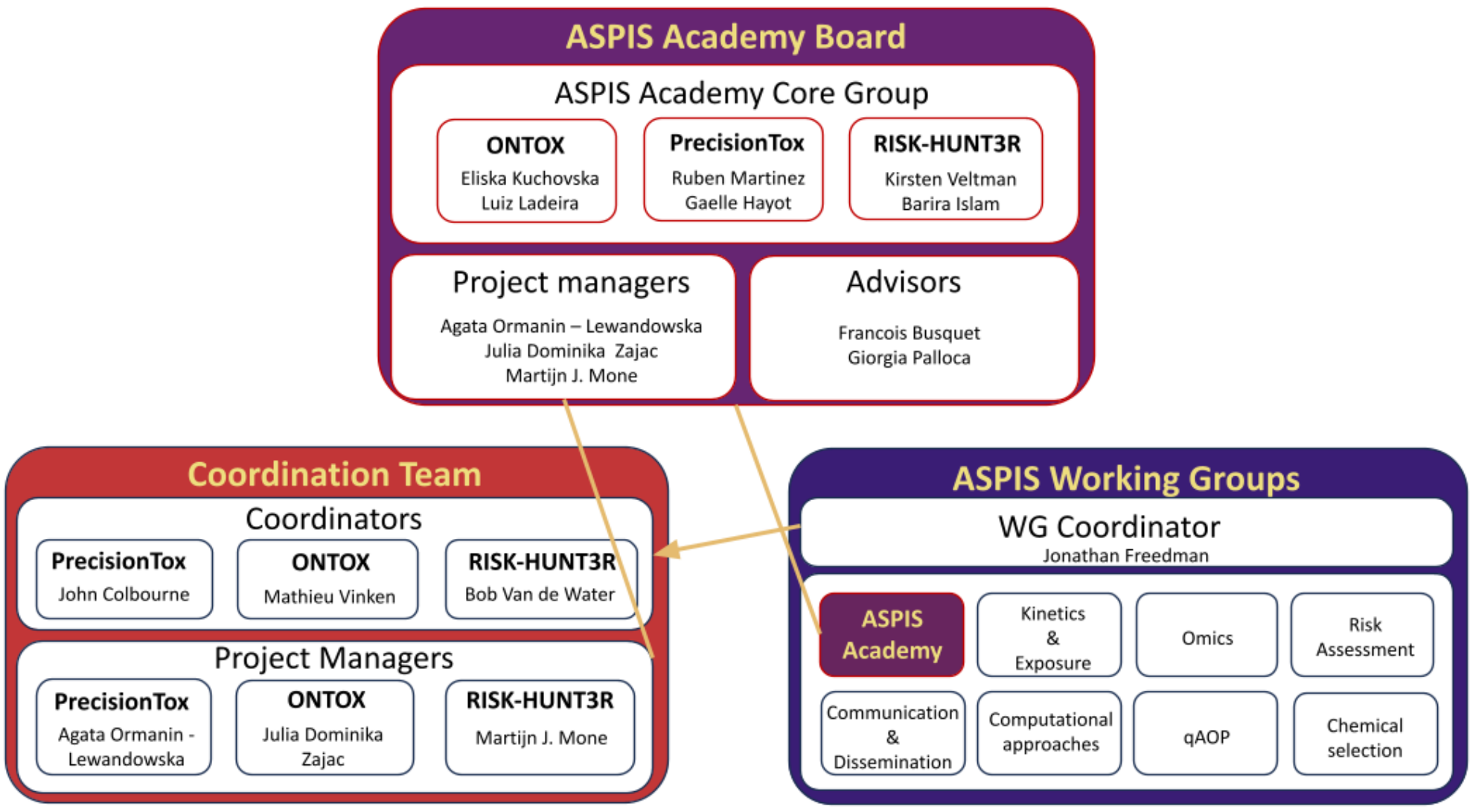

TERMS OF OFFICE

(a) The Board will consist of:
   a. 1 Advisor per consortium
   b. 1 x Project manager per consortium
   c. At least 2 x ECR Representatives per consortium
(b) Membership is agreed via direct application to the Board, should a position be vacant.
(c) Requirements for new Board members
   a. Advisors: experience in leadership or advising of networks or teams, experience with ECRs is an advantage
   b. ECR representatives: enthusiasm

(d) Membership within the Board is of indefinite length, if (i) the member remains willing to contribute to the Board and (ii) the Board remains confident in the member's ability to contribute. Members can terminate their post by writing at any time (by e-mail or letter) to the other members of the Board.
(e) The role of the AA Board member is unpaid.
(f) Decisions will be reached collectively through a voting process, with each Board member holding one vote. In the event of a member's absence, the option exists to delegate their voting authority to another member. In cases of parity, the proposed course of action must undergo revision until a consensus is established among the differing parties.

The founding Board members are as follows:

Position	Name	Role/Duties
ASPIS Diss & Com WP chair	Francois Busquet	Advisor
ASPIS Diss & Com WP chair	Giorgia Pallocca	Advisor
Project manager for PrecisionTox	Agata Ormanin - Lewandowska	Link with coordination team
Project manager for ONTOX	Julia Dominika Zajac	Link with coordination team
Project manager for RISK-HUNT*3R*	Martijn J. Moné	Link with coordination team
ECR representative for ONTOX	Eliska Kuchovska	1. Coordination & reporting<br>2. Communication with AA members (+Gmail administration)
ECR representative for ONTOX	Luiz Ladeira	1. C&D activities<br>2. Communication with external people
ECR representative for RISK-HUNT*3R*	Barira Islam	1. Mentoring program<br>2. Coordination & reporting
ECR representative for RISK-HUNT*3R*	Kirsten Veltman	1. Communication with external people<br>2. managing programs
ECR representative for PrecisionTox	Ruben Martinez	1. C&D activities<br>2. Managing trainings
ECR representative for PrecisionTox	Gaelle Hayot	1. Managing trainings<br>2. communication with AA members (+Gmail administration)

DUTIES

The Board will support the development of the ASPIS Academy activities. To reach this goal, the Board will:

(a) Provide an effective and sustainable communication channel for ECRs
(b) Support ECR-led activities such as webinars and face-to-face meetings throughout the project, including special symposia and poster sessions at cluster meetings
(c) Help grow the careers of ECRs and create job opportunities
(d) Organise social and professional networking events

(e) Create a support group for ECR

Specific duties of every board member are listed in the table in Section two. Every duty is assigned to 2 core group members to ensure that the duty is taken care of in case of illness or high workload. The communication of AA activities towards its members or the members of the three projects is done on a rotating basis so that all AA ECR representatives make their name and the invitations are sent by the representatives of the three projects.

2.3 **Meeting frequency**

(a) The Core Group will meet at least monthly. Additional meetings can take place if required. AA Board meetings will take place bi-monthly.
(b) Meetings will occur primarily via telecommunications; however, in-person meetings will take place during the ASPIS Open Symposiums.
(c) Meetings should take no more than one hour.
(d) Minutes of the meetings will be made available to all the members of the Board.

2.4 Reporting requirements

The AA Board will feed into ASPIS reporting requirements where appropriate. In particular, the AA Board will contribute to the ASPIS annual report, report to the ASPIS coordination team during Working Group Chairs meetings, and report on the ASPIS Academy activities from the perspective of 3 projects in the periodic reports. Jonathan Freedman (ASPIS coordinator) will be invited to regular AA Board meetings.

2.5 **Management**

The AA Board reports directly to the ASPIS Coordination Team.

This Board's Governing Document is subject to review every 18 months.

→ First version launched in December 2023
  - Next revision ongoing in Summer 2024

3. COMMUNICATION PLAN

This document describes the **communication and dissemination (C&D) strategy** of AA and sets out the overarching communication and dissemination strategy for the 1st year of the AA (the year 2024). This document will be updated at the end of 2024 (or sooner if needed) to include a C&D plan for the years to come based on the experience and feedback gathered in 2024. As such, the C&D plan identifies and outlines the overall communication and dissemination strategy of the AA and the roles of the AA Board and AA members, determines the internal communication and information sharing within AA, provides key messages to the targeted audiences to ensure that AA reaches its objectives and delivers its expected impact, outlines the evolving progress of the plan and provides a schedule for the C&D activities, and tools for their monitoring.

3.1 Communication & dissemination strategy & target audiences

Communication strategy

Communication strategy includes:

1. **Internal communication** of AA Board, communication between AA Board and the ASPIS coordination team, and communication towards AA members.
2. **External communication** of AA envisaged to engage the (expert and non-expert) public. The communication strategy is aimed both to enhance the competitiveness of the ECRs in view of new career perspectives and to boost the wider impact of the research of the ECRs on potential users.

1. Internal communication

AA Board communicates whenever needed via mail or WhatsApp group. The aspisacademy@gmail.com is added to recipients when communicating with AA members or external speakers, etc. for tracking purposes. Outgoing conversations are carried out using the institutional mail accounts of one of the AA Core group members because the Gmail may not pass the firewall. Gmail account is, however, used for incoming mails (e.g. AA members asking for advice). Managing the Gmail account is one of the duties of the AA Board, assigned in the governance section.

Communication from the AA Board towards AA members is carried out via mail and/or via the AA LinkedIn group https://www.linkedin.com/groups/9370153/. The communication frequency depends on the planned activities and current information-sharing needs. The mailing list of the AA members is regularly updated and the ECR lists of each project are the responsibility of the respective project AA core group members. *Link redacted.* Only active members of the three projects are kept on the ECR lists during the first year of AA (2024).

Active members are defined as ECRs being employed or having an internship in one of the three projects. Their contact is moved to an alumni list after the termination of their employment or internship. The creation of an alumni network will be decided and defined in the upcoming updates of this document. AA members should be encouraged to add their personal mail address to the list if they plan to leave ASPIS soon and wish to stay in contact with AA. The creation of a 2nd mailing list including alumni and eventually external ECRs will be discussed after the 1st year of AA operation.

The **ASPIS ECR Great Galaxy** (ASPIS-EGG) is a document containing contact information of AA ECRs and serves to facilitate the networking of ECRs and getting to know each other. Currently, the document is in preparation and is stored at the AA Gmail drive account on the following link:
link redacted

Interim ONTOX document:

link redacted

Interim PrecisionTox document:

link redacted

Interim RISK-HUNT3R document: does not exist

Reporting

AA Core group attends the ASPIS Communication and Dissemination WG and reports on the AA activities. AA Core group ONTOX members join the ONTOX communication and dissemination WP meetings and AA Core group RISK-HUNT3R members may join RISK-HUNT3R communication and dissemination meetings ad hoc (there are no such meetings in PrecisionTox). AA Core group is also responsible for reporting to the ASPIS Working Group Chairs meeting once per month, this duty is assigned to one member in the governance section.

2. External communication

During the first year of AA's operation, a better-defined plan for AA's outreach and activities to the general and professional public will take shape. The initial activities of the 1st year of AA operation include the creation of short videos by AA members and/or the creation of a blog containing short entries from AA members.

Moreover, potential sponsors of AA activities identified during C&D activities are entered into a spreadsheet for possible future funding opportunities. *link redacted*

Furthermore, **offline communication** (both internal and external) includes face-to-face meetings throughout the project, including special symposia and poster sessions at cluster meetings, summer schools, ad hoc meetings at conferences, and other opportunities.

Target audiences

AA' target audience is first and foremost the ECRs of the three projects. When AA is opened to external members (this possibility will be discussed at the end of 2024), the target audience will be composed not only of ASPIS ECRs but also of external ECRs from other NAM-related or even non-NAM-related projects.

The target audiences of the communication and dissemination outcomes produced by the AA ECRs (short videos, blog entries, …) are the identified audiences of the three projects.

3.2 Communication & dissemination tools & activities

Project logo

For ease of identification, AA has adopted a logo (Fig. 1). The logo must be used for any external activity, website, brochures, and press materials related to AA. It is not necessary to include it in internal mail communication.

Fig.1: ASPIS Academy logo

ASPIS Academy Starter Pack

All new members of AA will receive at the beginning of AA operation an introductory pdf document containing all the necessary information:

- Link to subscribe to the mailing list and agree with mail address use and internal sharing
- Link to the ASPIS ECR Great Galaxy
- Link to the LinkedIn group including its purpose and reminder to actively participate
- Link to the ASPIS folder on SharePoint to share recordings of the meetings, etc.
- Description of the AA programs
- Link to an anonymous suggestion form
- Mail contact to AA aspisacademy@gmail.com
- Contact details to ASPIS WG chairs including a reminder for ECRs to join the WGs, and link to the ASPIS website with WG descriptions

The document is currently in preparation and can be accessed on the aspis academy google drive on the following link. *Link redacted, however, the final Starter Pack is available in Supplemental File 3 of this publication.*

Activities

AA communication & dissemination activities are summarized in Table 2. Other non-C&D activities (AA programs) are described in section 4 of this document. The main activities are internal online meetings for AA members organized by the AA core group and short videos and blog entries written by the AA members themselves. AA core group decided not to limit our activities to English-speaking EU citizens, thus the short videos and blog entries would contain subtitles in all languages used in the three consortia. Altertox will organize a short training webinar for ASPIS ECRs focused on video creation and will provide advice for any technical, design, or concept difficulties.

Table 2: ASPIS Academy communication & dissemination activities

Activity	Details	Frequency	Organizer / Author	Target audience
**Online meetings with guests**	Topics: career development, networking, soft skills	1x per 2 months	AA Board	AA members
**Short videos**	About the work of ECRs	3 (1 per each project) once per 3 months	AA members	Expert public, ECRs, audiences of the 3 projects
**Blog entries, memes**	About the work of ECRs	3 (1 per each project) once per 3 months	AA members	Expert public, ECRs, audiences of the 3 projects
**Summer school**	Networking and training	1x per year	AA Board	AA members

Templates and examples of the short videos and blog entries with memes will be prepared by the AA Core group.

A calendar of the planned online meetings with guests is available at *link redacted*.

Another dissemination activity to consider is writing a publication authored by the AA core group based on our AA experience of the year 2024 containing “Survival hacks for ECRs”.

Potential collaborations with other ECR networks

- YoungTPI
- VHP4Safety young scientists
- Marie Curie Academy
- Eurotox Early Career Forum
- PARC ECRs

3.3 Communication & dissemination channels

AA LinkedIn group serves as an internal channel to inform members about AA news such as meetings but also to share interesting and relevant external webinars, conferences, funding opportunities, summer schools, etc. Members should be periodically motivated by the AA core group to engage in sharing such information with fellow members of AA.

Channels for C&D towards external audiences include the ASPIS webpage and webpages of the 3 projects as well as social media of the three projects (LinkedIn, Twitter). The blog entries will be published on the ASPIS webpage and shared on social media. AA short videos will be uploaded to one of the three projects YouTube channel and shared via social media.

The newsletters of the three projects will host snapshots of the blog posts and videos created by AA members. AA Core group will consider adding and creating an Instagram account to the channels.

AA meetings and webinars will be organized via Teams or Zoom software and will be recorded and made available to ASPIS ECRs on the ASPIS SharePoint.

3.4 Sustainability plan

The sustainability plan will be established at the end of 2024 after gaining experience and feedback on AA activities during the year 2024.

4. PROGRAMS

4.1 ASPIS Academy Mentoring Program

The ASPIS Academy Mentoring Program is a dynamic initiative designed to empower emerging professionals within the field of New Approach Methodologies (NAMs) by providing structured and personalized mentorship, fostering professional growth, and contributing to the development of well-rounded, skilled professionals. This program is committed to creating a supportive community within ASPIS who are dedicated to the advancement of knowledge, expertise, and careers in the field of NAMs in toxicology. Mentors and mentees will work together to build a path to success, innovation, and excellence in toxicology.

Program Goals

- <u>Knowledge Building</u>: Facilitate the building of knowledge, skills, and experiences from the guidance of experienced professionals to ECRs and from senior ECRs to junior ECRs.
- <u>Professional Growth</u>: Support mentees in setting and achieving their career development goals, which may encompass research, publications, presentations, certifications, and other professional milestones.
- <u>Networking</u>: Expand the professional networks of both mentors and mentees, connecting them to a broader community within the ASPIS Academy and the field of toxicology.
- <u>Career Advancement</u>: Enhance career prospects for mentees by offering guidance, advice, and resources that help them excel in their chosen career paths.

Program Structure

- <u>Career planning session</u>: Before the one-on-one mentoring starts, the mentees will have the opportunity to attend a career planning session and learn how to structure their career goals and align with their learning paths. This will help identifying the skills that need guidance, aiding the matching process to their mentors.
    - The career planning session might be coupled with the ASPIS Open Symposium, or any other meeting in which we are expecting attendance of ASPIS ECRs. The session should be open to everyone and as general as possible to meet the needs for general aspects of career planning. Personalized issues can be addressed in a second moment by demand.
- <u>Mentorship Pairs</u>: Each mentor will be matched with a mentee, cultivating a one-on-one relationship for personalized guidance and support. This is not intended to overlap with the PI roles. The mentees should choose a mentor based on topics or skills listed on the mentor profile they would like to talk about.
    - During the career planning session, the Mentoring Program will be launched, and forms will be made available to ECRs and senior scientists within the cluster for registration. The mentee form must contain contact information, affiliation, relevant links for the affiliation labs, LinkedIn profile, a list of skills that the ECR wants to develop aligned with the planning made at the career planning session and topics of interest. The mentor form must contain contact information, affiliation, relevant links for the affiliation labs, LinkedIn profile and a list of topics that the mentor could provide mentorship. ECRs can also volunteer as mentors.

- Duration: The mentoring relationship will span the timeframe defined by the mentor and mentee based on their planning; this could take any duration, from one session to more than a year, with regular meetings.
- Mentoring Activities: Pairs will participate in activities such as regular meetings, goal-setting sessions, career planning, and knowledge-sharing discussions. The format and frequency of these activities will be customized to meet the needs and availability of the mentor and mentee.
- Program Support: ASPIS Academy will provide mentors and mentees with profile descriptions, guidelines, and support to ensure they make the most of their mentoring relationship.

Program Benefits

- Mentors: a) Opportunity to share knowledge and expertise, making a significant impact on the next generation of toxicologists. b) Fulfillment derived from contributing to the professional development of emerging professionals. c) Expansion of the professional network and fostering lasting connections.
- Mentees: a) Access to valuable insights and guidance from senior professionals. b) Personalized support for realizing career goals and reaching new heights. c) Enhanced networking opportunities within the field of toxicology.

Evaluation and Feedback

- Periodic evaluations will be conducted to assess the effectiveness of the mentoring relationship and make necessary adjustments.
- Continuous feedback from both mentors and mentees will be encouraged to ensure the program's continued improvement and success.

We recognize that having a mentor who is not the mentee's PI may cause contradictory sentiments, and if this is a big concern for the program's feasibility, we have a second approach. Instead of one-on-one mentoring, we may establish it as a career development program. In a career development program, we can have a career development specialist chairing periodic meetings and assisting ECRs in establishing their own objectives in a short-, mild- and long-term career plan.

4.2 ASPIS Academy Twinning Program

The ASPIS Academy Twinning Program will involve matching ECRs wishing to master a specific research skill or technique with an experienced host. ASPIS Academy will inventory opportunities and requests and accelerate trainee-host pairing using the ASPIS Academy ECRs Great Galaxy (ASPIS-EGG) document, containing concise and concrete profiles of participating laboratories and companies. Additionally, ASPIS Academy will organize, in collaboration, open days at partner companies where several ECRs get the opportunity to be shown around and experience the working environment. The Twinning Program will span the timeframe agreed upon between the host and trainee, and the financial responsibility lies with the respective institutions. Through the ASPIS Academy Twinning Program ECRs gain the opportunity to build knowledge and skills from guidance by experts and obtain experience in other work environments.

Program goals

- Knowledge and expertise sharing: Facilitate the building of knowledge, skills, and experiences from the guidance of experienced professionals to ECRs/Support ECRs in mastering new skills and techniques.
- Accelerate collaborations: Advance contact between ECRs and experienced hosts working on similar topics or techniques.

Program structure

- Program support: ASPIS Academy will inventory "supply" and "demand" with concise and concrete profiles of opportunities and requests, and make sure that this is frequently updated. The ASPIS ECR Great Galaxy (ASPIS-EGG) document will serve as a basis for trainee-host pairing. Moreover, ASPIS Academy will serve an accelerating role in communication between ECRs and experienced hosts.
- Company open house: ASPIS Academy will organize, in collaboration, open days at partner companies where several ECRs are shown around and experience the working environment.
- Duration: The Twinning Program will span the timeframe agreed upon between the host and trainee.
- Finance: The Twinning Program is financed by the respective institutions.

4.3 ASPIS Academy Mirroring Program

The mirroring activity will involve matching the ECRs, based on their interest and experience, to the initiatives in ASPIS. There are eight working groups (WGs) currently running under ASPIS umbrella. A comprehensive description of the WGs is available at the following link: https://aspis-cluster.eu/working-groups/. For the success of WG activities, it is important to make ECRs aware of these WGs and encourage their participation in WG meetings. Most importantly, attendance of the WG meetings and active participation on their work will allow the ECRs to learn from senior researchers outside of their team. ASPIS Academy can serve as an important link between the ECRs and WGs. To initiate mirroring activity, ASPIS Academy will match the ECRs with the WGs and inform them of the WG initiatives. A session for this purpose is being organized to be held online in December at the 1st ASPIS Academy meeting. ASPIS members will also receive information about the WGs in the ASPIS Academy Starter pack. Moreover, ASPIS Academy members have started filling out an ASPIS ECR Great Galaxy document. This document contains a short summary of the background and interests of ECRs. The ASPIS ECR Great Galaxy and the mirroring activity will also be of use for activities beyond ASPIS where a contribution from ASPIS partners is required.

4.4 ASPIS Academy Training Program

ASPIS Academy Training Program goal is to build and enhance the formation of the ECRs in different needs which will be necessary for their future scientific career. Those needs include several aspects and fields, including, for example, both soft and hard skills. Experienced professionals will guide and train the ECRs in both NAM-related and transversal new skills, techniques, knowledge, etc. ASPIS Academy Training activities will be based on the needs of the ECRs and will be supported and organized by the three member projects, both as webinars and as on-site activities during consortium/congress presential meetings.

Program trainings:

- Soft skills: this set of trainings will cover knowledge and skills that ECRs could generally apply in their career, and that are not necessarily related with NAMs, e.g.:
  - General science communication
  - Building and maintenance of a scientific network
  - Publication writing (both scientific and dissemination articles)
  - Grant writing
- Hard skills: this set of trainings will be mainly focused on NAMs and experimental/ computational techniques, e.g.:
  - NAMs trends and new techniques/future of NAMs (lectures)
  - Data treatment and graphics
  - Introduction to R software/machine learning
  - NAMs' techniques (e.g., omics, QSARs, etc.)

Past trainings' examples:

- Webinars:
  - 8 webinars (PrecisionTox Café scientifique, https://precisiontox.org/cafe-scientifique/) and 5 podcasts (PrecisionTox Voxlab, https://precisiontox.org/voxlab/) about *in vitro* and non-sentient organisms categorized as NAMs.

- On-site trainings:
  - ASPIS training on Science Communication (ASPIS open symposium meeting, 24.11.2022)
  - Applications for Artificial Intelligence (AI) in Risk Assessment (ASPIS open symposium meeting, 25.11.2022)
  - ASPIS Academy training: Grant writing masterclass (ASPIS open symposium meeting, 15.09.2023)

Program structure

- Schedule: the calendar will adapt to the future webinars and on-site trainings planned by the three member projects.
- AA role: ASPIS Academy will help in the collection of the training needs from ECRs to help and propose new trainings to be performed which would cover the formation "demand" from ECRs.
