# Supplementary material for: ASPIS Academy: A blueprint for building early-career researcher networks to advance NAM-based risk assessment
Source: NAM J. 2026 Aug 7;2:100124. doi: 10.1016/j.namjnl.2026.100124 (PMC13482515; doi:10.1016/j.namjnl.2026.100124)
Supplement: Supplementary file 3 [file mmc3.pdf]

ASPIS ACADEMY

STARTER PACK :: ASPIS ::

A science network for the development and usage of non-animal new approach methodologies

aspis-cluster.eu/aspis-academy/

The **ASPIS Academy** is a network for early-stage researchers (ESRs) who are into using New Approach Methodologies (NAMs) in toxicology. We're all about boosting the careers of ESRs by giving them specific training, making sure everyone gets a fair shot, and setting up a space where the awesome ideas and dreams of a new generation of young scientists can shine. Join us and let's make NAMs a lot more fun!

ONTOX — ontox-project.eu

RISK HUNT3R — risk-hunt3r.eu

PRECISION TOX — precisiontox.org

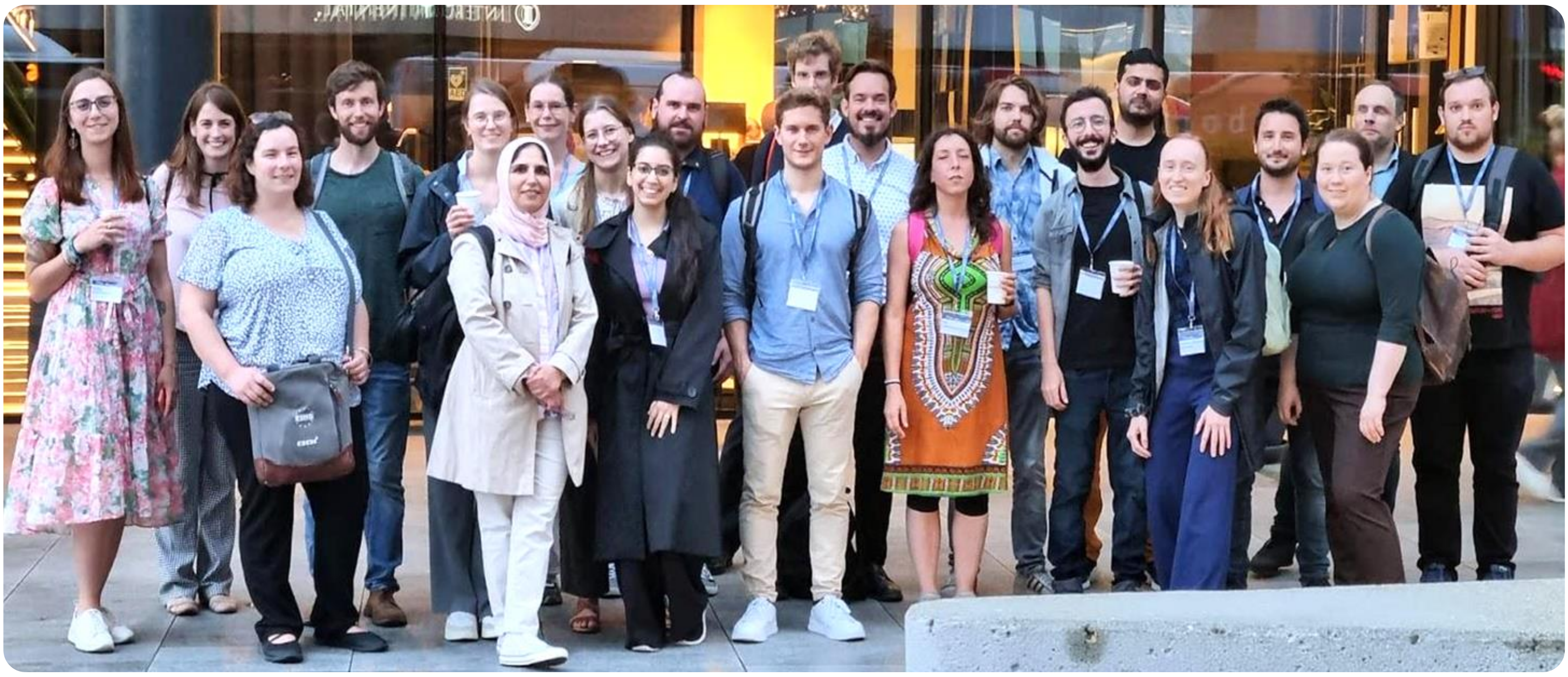

Who are our ESRs?

At the **ASPIS Academy**, *"early-stage researchers (ESRs) are defined as researchers who received their last education diploma not more than 10 years ago. Researchers working both in academia and industry as well as master and bachelor students are included."*

If you are not listed in our mailing list, please contact us at aspisacademy@gmail.com

ASPIS ACADEMY

Get involved!

We are planning a lot of cool activities and programs for you to get involved, including opportunities of research stays in the ASPIS involved laboratories, a mentoring program for career development and much more! - You can find a full description of the programs HERE. You can express your interests HERE.

Communication task force: Help us producing outreach content: videos/blog posts/memes.

Training program: Several workshops and webinars at the ASPIS Open Symposium and online.

Twinning program: Student lab exchange.

Mirroring program: Linking individual ASPIS WGs with ASPIS Academy.

Mentoring program: One-to-one relationship with experts within ASPIS from industry, regulatory agencies and academia.

Summer School: in-person 2-3 days long summer school focused on risk-assessment using NAMs.

Stay tuned!

Here is a collection of useful resources to start at the ASPIS Academy!

ASPIS Academy Great Galaxy: information on all your ESRs colleagues and you! Include yourself if you are not there yet, this will make easier to network and find people with common interests.

ASPIS Academy LinkedIn group: this is the Academy group, to share interesting stuff related to NAMs and science. This is our open space, so feel free to use it!

ASPIS Academy webpage: you will find more information on what we are doing, our programs descriptions, how to get involved, and much more.

For any question or comment, please contact us at aspisacademy@gmail.com or use our anonymous contact form HERE.

Join the **ASPIS Working Groups**:

Collaboration groups between ASPIS consortia are organized as working groups. Joining a WG as an ESRs is a unique opportunity to learn more about your topic of interest with senior researchers and participate actively in the discussions.

1. Chemical selection;
2. Risk Assessment;
3. qAOPs;
4. Omics;
5. Kinetics and exposure;
6. Computational approaches;
7. Communication and dissemination;
8. Database.

More info at: https://aspis-cluster.eu/working-groups/ or contact one of the WG chairs HERE.

These projects have received funding from the European Union's Horizon 2020 research and innovation programme under grant agreement
No. 963845 (ONTOX),
No. 965406 (PrecisionTox),
No. 964537 (RISK-HUNT3R).
This output reflects only the author's view and the Directorate-General for Research and Innovation (DG RTD) cannot be held responsible for any use that may be made of the information contained therein.

https://aspis-cluster.eu/

ONTOX RISK HUNT3R PRECISION TOX
