# Supplementary material for: ASPIS Academy: A blueprint for building early-career researcher networks to advance NAM-based risk assessment
Source: NAM J. 2026 Aug 7;2:100124. doi: 10.1016/j.namjnl.2026.100124 (PMC13482515; doi:10.1016/j.namjnl.2026.100124)
Supplement: Supplementary file 4 [file mmc4.pdf]

ASPIS Academy: A Blueprint for Building Early-Career Researcher Networks to Advance NAM-Based Risk Assessment

SUPPLEMENTAL FILE 4 – ANNUAL ASPIS REPORTS – only the ASPIS Academy chapters are provided in this supplemental file. For the full reports, visit the ASPIS webpage *https://aspis-cluster.eu/annual-reports/*.

The ASPIS Academy was one of the ASPIS thematic working groups (WGs, **Supplemental Figure 1**), including Chemical Selection, Risk Assessment, quantitative Adverse Outcome Pathways (qAOPs), Omics, Kinetics and Exposure, Computational Approaches, Database, Communication and Dissemination, and the ASPIS Academy. These WGs brought together investigators from the three consortia to address key scientific and organizational challenges relevant to the cluster's mission. Early-career researchers from the ASPIS Academy were able to participate in the other WGs aligned with their interests and contribute to cross-consortia activities through the Mirroring Program. In 2025, ASPIS transitioned from a WG-based structure to a task force-based model (**Supplemental Figure 2**). While most WGs were discontinued, the ASPIS Academy remained active until the end of the ASPIS projects in October 2026.

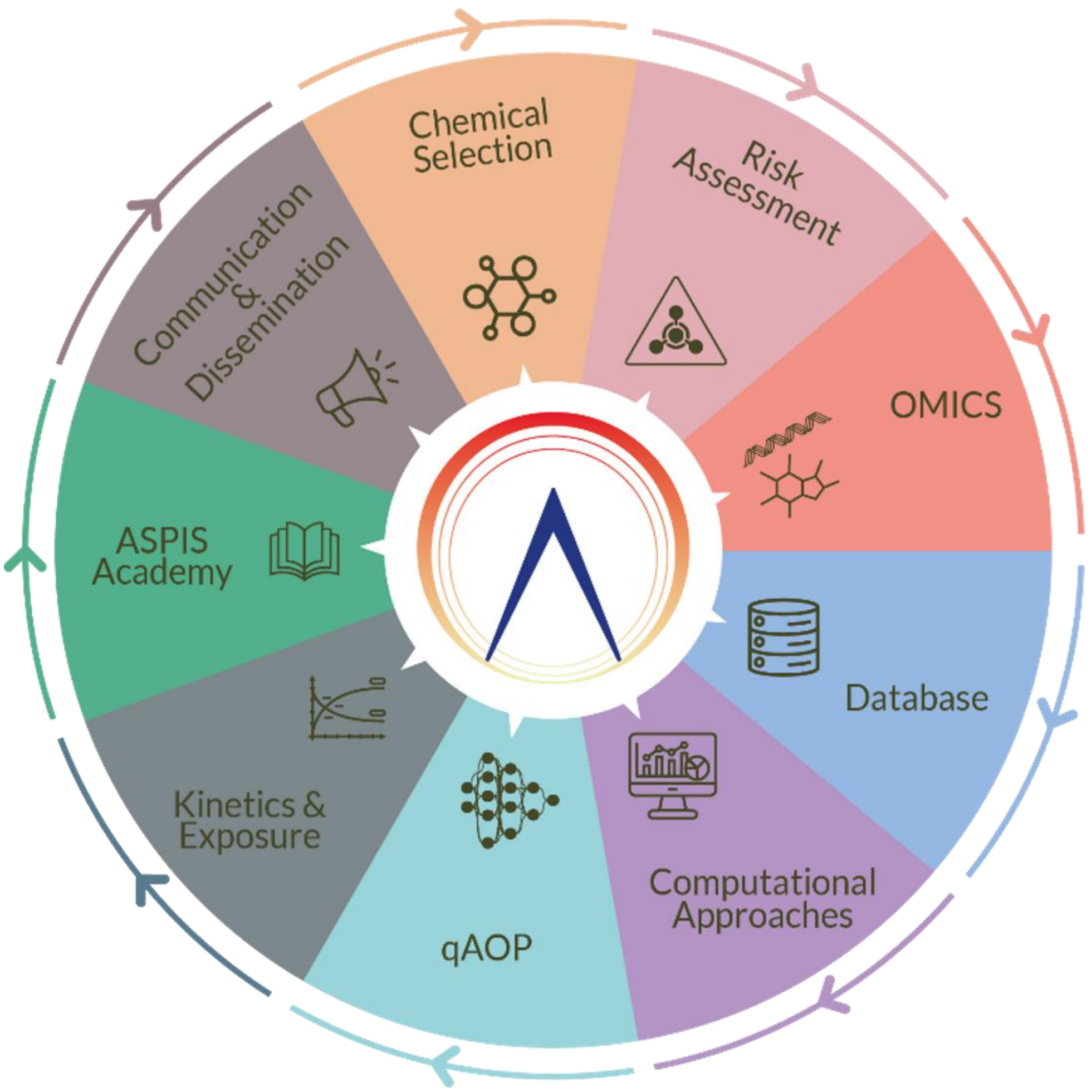

Supplemental Figure 1: ASPIS working groups (WGs).

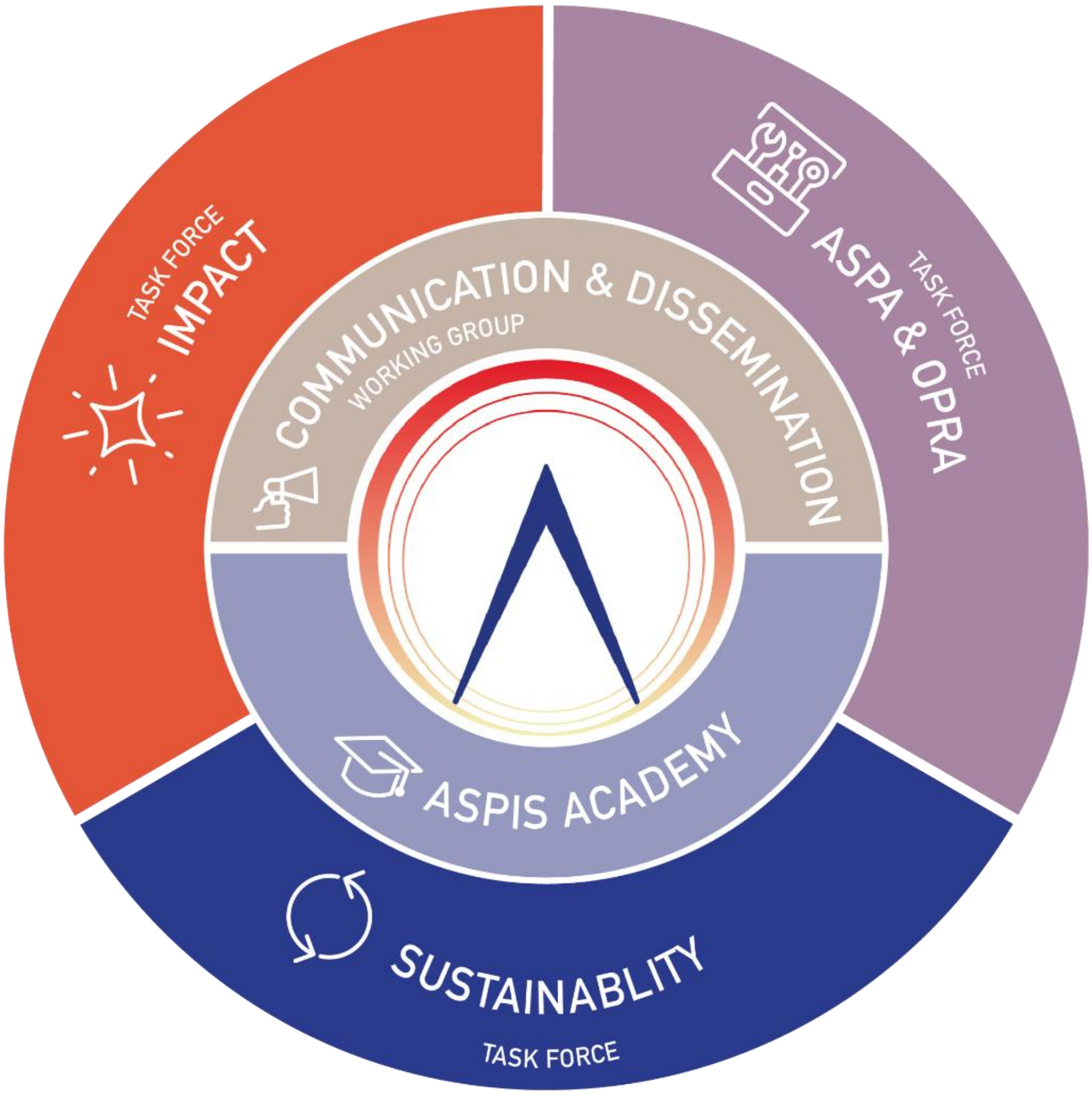

Supplemental Figure 2: ASPIS task forces.

SECOND YEAR ANNUAL REPORT FOR ASPIS

Individual Working Group Activities

ASPIS Academy Working Group

Co-chairs: Eliska Kuchovska (ONTOX), Luiz Ladeira (ONTOX), Barira Islam (RISK-HUNT*3R*), Kirsten Veltman (RISK-HUNT*3R*), Ruben Martinez (PrecisionTox), Gaelle Hayot (PrecisionTox)

Background

The purpose of the ASPIS Academy (AA) is to build a viable early-stage researchers (ESRs) network focused on the use of New Approach Methodologies (NAMs) for chemical risk assessment. The ASPIS Academy promotes the careers of ESRs by providing specialized training, promoting equal opportunity for all the members, and creating a platform devoted to the voices and aspirations of a new generation of young scientists who will become natural experts and future leaders of NAMs and their applications.

During the 2nd half of 2023, the AA Core Group was set up, comprising 2 ESR representatives of each project. The Core Group is completed by two advisors and three project managers forming the ASPIS Academy Board, the governing body of the AA. The duties of AA Board members and reporting requirements to the ASPIS C&D WG, respective projects, and the ASPIS coordinator are described in the drafted AA Governance section in the AA document in the annexes and illustrated in the figure below. Moreover, the definition of AA ESRs was formulated as *researchers who received their terminal education diploma not more than 10 years ago. Researchers working both in academia and industry as well as master and bachelor students are included. Exemptions may apply if event capacity and budget allow it.* Currently, AA ESR membership is restricted to the ASPIS cluster.

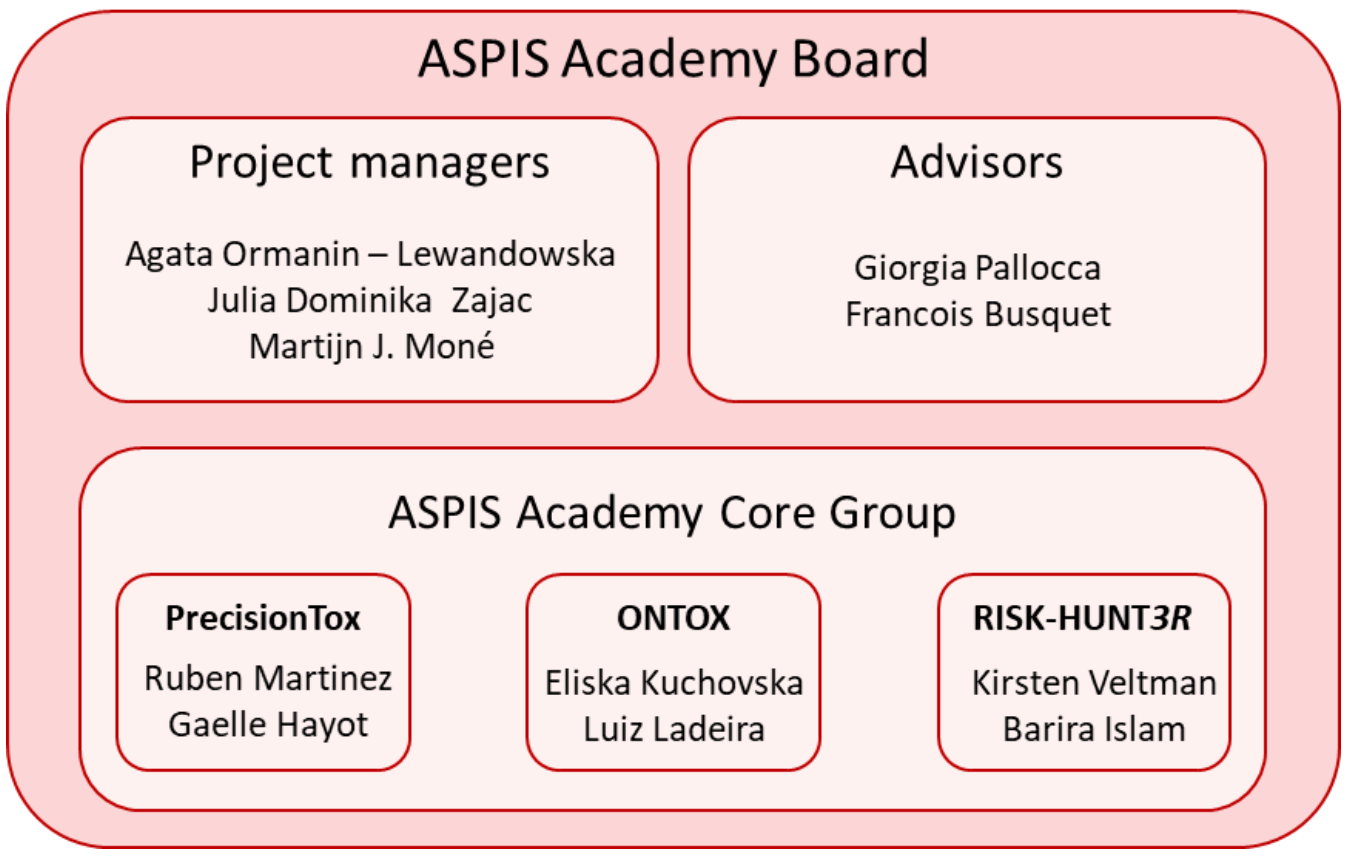

The planned activities for AA members include a mentoring program (one-on-one mentoring from senior mentors to ESRs but also from senior ESRs to junior ESRs), a twinning program (ESR visits at hosting institutions, open days at companies), a mirroring program (matching ESRs with other ASPIS WGs), and training program (webinars and on-site trainings). All activities will start at the beginning of 2024 except for the already ongoing training activities. Moreover, a communication plan was drafted describing the strategy, target audiences, channels, tools, and C&D activities and is attached in annexes. This communication plan was created based on the ONTOX communication plan.

Successes and Opportunities

The AA held one on-site training for the ESRs entitled Grant Writing Masterclass at the ASPIS Open Symposium in September 2023 in Ljubljana, Slovenia. On the same occasion, a networking sightseeing event was organized by members of the ASPIS Academy themselves. The AA inaugural meeting is planned for the middle of December 2023 when the AA Core Group will introduce the AA aims, structure, programs, and plans to ASPIS ESRs. Furthermore, AA Core Group created an AA LinkedIn group as a networking online platform for ASPIS ESRs.

Shortcomings and Uncertainties

The AA activities (especially the summer school and twinning program) are dependent on budget opportunities which are scarce. Thus, the scope and number of activities will rely on potential financial support from external sponsors.

Aims for 2024

AA Core Group plans to organize several online trainings beginning with a career development session on 25.1.2024 inviting 4 guests from different backgrounds (academia, industry, intergovernmental organization, and NGO) to enable a discussion with ESRs. The organization of the first AA summer school is under discussion and would probably be coupled with another ESR network. Moreover, the AA Core Group will organize a poster session for the ESRs as well as on-site training at the ASPIS Open Symposium held in September 2024 in Copenhagen. The AA Core Group also plans to initiate an ESR C&D activity consisting of the creation of short videos/articles/memes to reach target audiences as described in the AA Communication plan, being able to also actively involve other member ESRs in AA coordinated actions.

Third Year Annual Report for ASPIS

(Reporting Period: November 2023 to November 2024)

ASPIS Academy (AA)

Co-chairs: Eliska Kuchovska (ONTOX), Luiz Ladeira (ONTOX), Barira Islam (RISK-HUNT*3R*), Kirsten Veltman (RISK-HUNT*3R*), Ruben Martinez (PrecisionTox), Gaelle Hayot (PrecisionTox), Shaleen Glasgow (PrecisionTox), Julen Sanz Serrano (ONTOX), Marie Corradi (ONTOX), Peter Pobis (ONTOX), Rita Ortega Vallbona (ONTOX), A. Melina Steinbach (RISK-HUNT*3R*), Eike Collen (RISK-HUNT*3R*) and Hiba Khalidi (RISK-HUNT*3R*).

Background

The ASPIS Academy (AA) is a network for early-stage researchers (ESRs) focused on the development and use of NAMs for chemical risk assessment. The AA was established in the second half of 2023 with the goal of promoting the careers of ESRs by providing specialized training and equal opportunities for its members and creating a platform devoted to the voices and aspirations of a new generation of young scientists who will become experts and future leaders of NAMs and their applications. The AA is led by the AA core group composed of ESRs representing the three projects and advised by AA Board composed of the three ASPIS project managers (Agata Ormanin-Lewandowska - PrecisionTox, Julia D. Zajac - ONTOX and Martijn J. Moné - RISK-HUNT3R) and AA advisors (François Busquet, Helena Kandarova, Silvia Tangianu).

Successes and Opportunities

The year 2024 has been very successful for the AA. Thanks to the numerous activities organized for ~ 130 ASPIS ESRs. Additionally, the AA has opened some of its programs (especially the online trainings) to external ESRs. They were invited to join the *AA LinkedIn group,* where they are informed about AA activities. The AA organized two career development sessions in 2024, inviting eight guests with different career paths to give insights into their roles. The first session involved guests representing academia, industry, intergovernmental organization and an NGO, while the *second session* featured a forensic toxicologist, senior publisher, consultant and senior project manager. Three additional online training sessions were organized focusing on the topics: *Connecting with Your Audience*, *Finding European Research Funding and Writing a Competitive Research Proposal* and *The Importance of Science for Policy and Science Communication for 21st Century Toxicology*. The AA also organized one in-person training at the ASPIS OS in Copenhagen featuring three speakers discussing *How to Transform Your Research into a Spin-Off Business* (Figure 3).

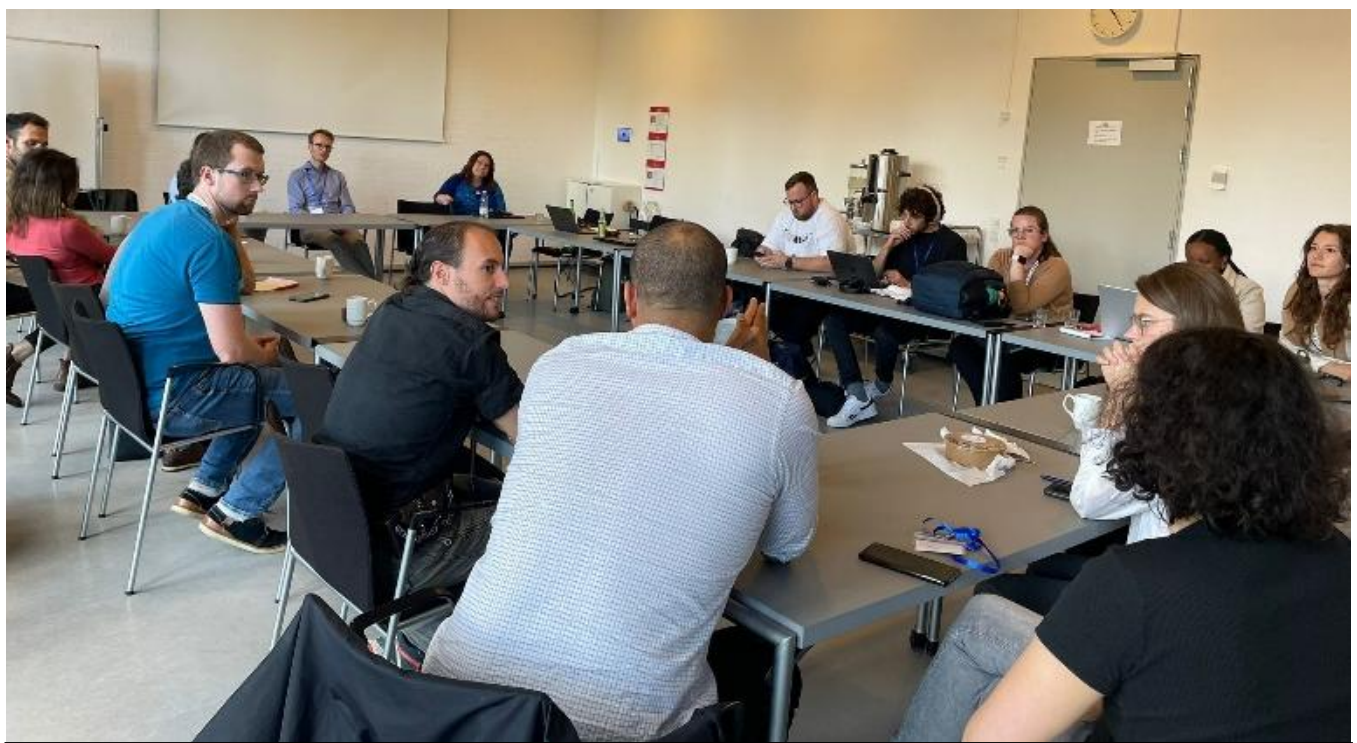

Figure 1. In-person training “How to transform your research into a spin-off business” at the ASPIS Open Symposium in Copenhagen in September 2024.

The ASPIS OS in Copenhagen allowed for more in-person activities including an AA poster session, presentation and networking activities including a sightseeing walk through Copenhagen and a pub quiz (Figure 4). Some of the trainings organized by the AA have been recorded and can be found on the *ASPIS YouTube channel.* The highlight of the year was a *scientific session organized by AA at the ESTIV congress* in Prague (Figure 5). The session

featured one presentation introducing the AA and three scientific presentations of selected ASPIS ESRs, one from each consortium.

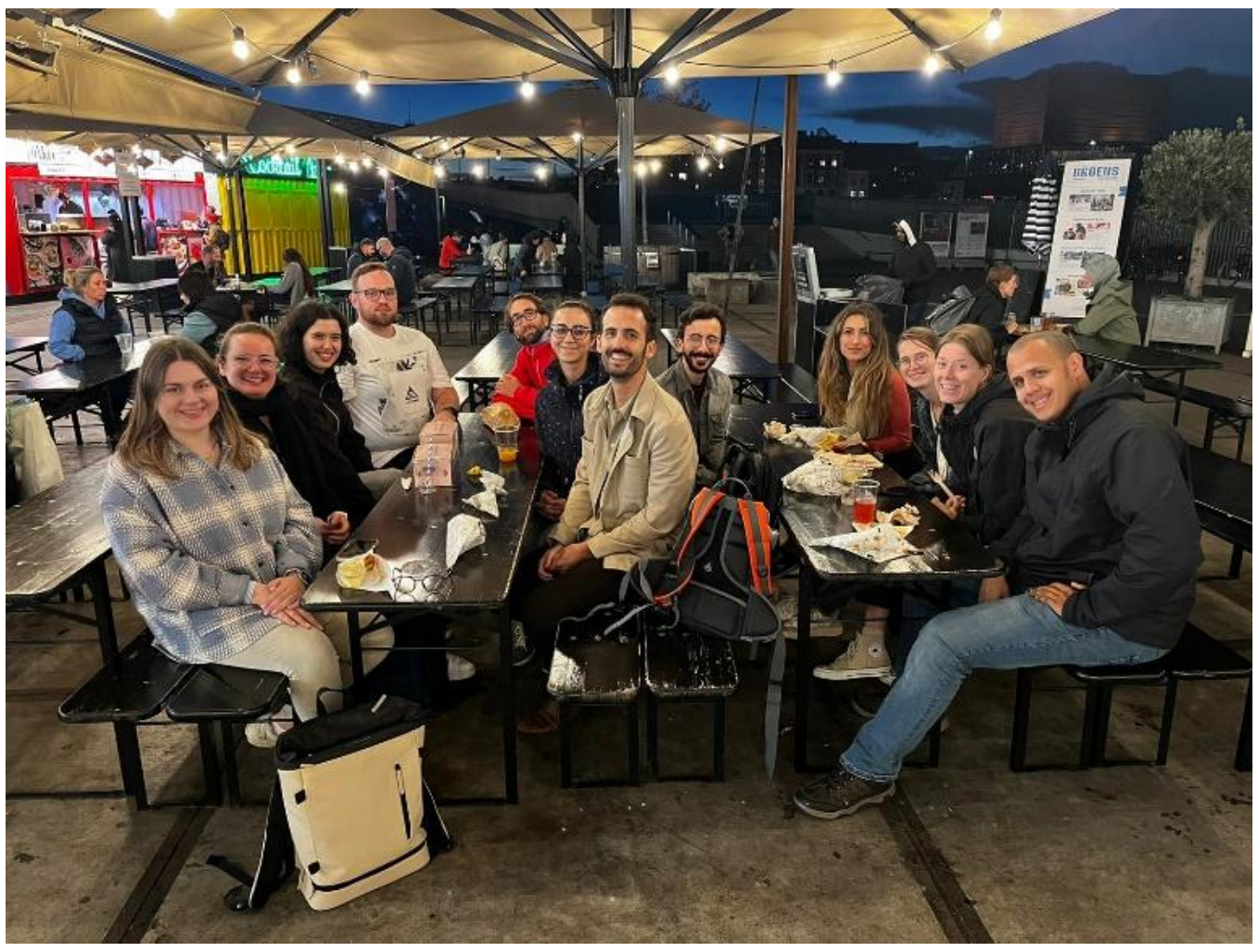

Figure 2. Networking evening of the ASPIS ESRs in Copenhagen

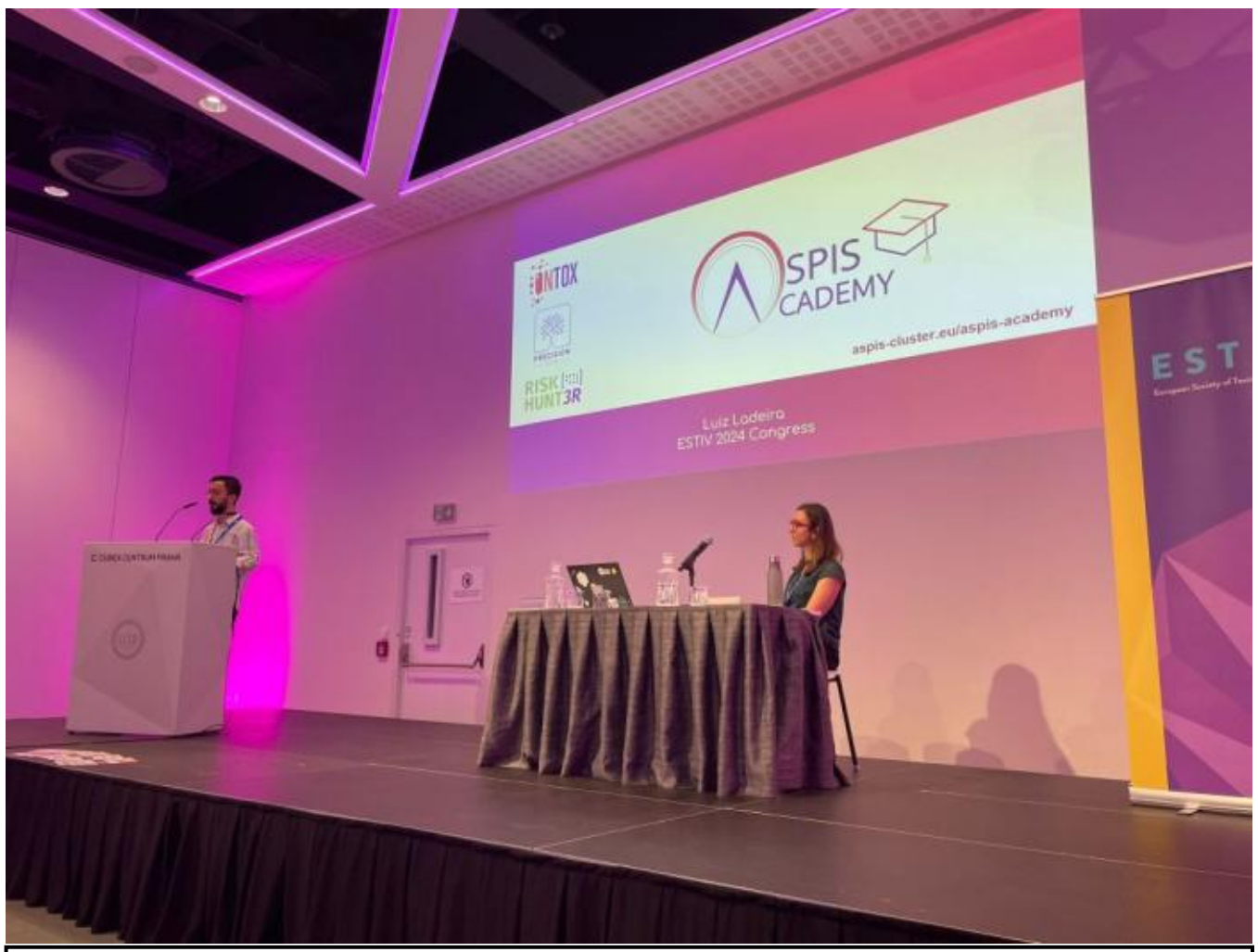

Figure 3. Scientific session organized by the ASPIS Academy at the ESTIV Congress in Prague in June 2024.

ESRs have been trained in conducting interviews of which *four were already published on the AA webpage*. The AA webpage has been extensively updated and expanded to include subsections about the *AA core group members*, *AA news* and a *subsection introducing the AA* featuring an introductory video. The AA has been working on its dissemination activities using various tools such as *videos*, posters at scientific conferences. The AA is also active at ASPIS and Altertox booths at conferences offering materials such as AA flyers, business cards and stickers. The AA has successfully carried out the first round of its mentoring program featuring ten mentorship pairs with the second round of applications planned for 2025. ASPIS ESRs joined the mirroring program bringing ESRs to ASPIS WGs to learn the necessary skills and obtain scientific and collaboration opportunities. The AA actively engaged in external collaboration opportunities by contacting and organizing joint activities with the *PARC Junior Community, young TPI, SETAC Student Advisory Council, ESTIV Early Career Network* and *EUROTOX Early Career Forum*.

Shortcomings and Uncertainties

AA activities are dependent on budget availability. Thus, the scope and number of activities rely on potential financial support from sponsors. Therefore, the Twinning program of the AA, which aims to facilitate short internships of ASPIS ESRs in ASPIS partner laboratories and companies, has seen only one ESR participating in this program.

Aims for 2025

Numerous activities are being prepared for 2025. First, the AA summer school is planned to take place in Valencia in April as a satellite event of the ONTOX annual meeting. Poster sessions, in-person training and networking activities are also envisaged for the ASPIS OS in Athens in September.

The AA submitted two session proposals for conferences: 13th World Congress on Alternatives and Animal Use in the Life Sciences and SETAC Europe. Additional AA sessions might take place in 2025 following the successful example of the ESTIV session in 2024. The AA plans to continue with its career development activities in the training, mentoring and twinning programs. Additionally, four interviews with AA speakers are now being conducted and will

be published in 2025. The AA core group is preparing a scientific publication about AA; its creation, goals and legacy with an expected publication date in the first half of 2025. The year 2025 will especially mark AA's focus on its sustainability and network legacy. Finally, in 2024, most of the original core group members left AA and new ESRs from the three projects joined the efforts (both original and new members are listed as co-chairs above). Their new fresh ideas might give rise to new additional AA activities.

Impact

The AA is contributing to the development of non-animal safety assessment approaches, aligning with the 3Rs principles, with a strong focus on 'Replacement'. The AA training programs help ESRs to develop and use innovative methods to replace animal-based approaches in safety assessments. AA also helps ERSs develop critical skills to align their work with regulatory demands, thereby encouraging the adoption of these approaches by industry and regulatory bodies. Finally, the AA focused efforts on sharing outcomes of ASPIS ESRs via different communication and dissemination tools and conference participation are designed to encourage the widespread adoption of developed methods, further amplifying their impact and commercial viability. Through these measures, the Academy's activities align with the goal of implementing sustainable and innovative safety assessment approaches.

Status

The AA will continue its activities.
