# Supplementary material for: ASPIS Academy: A blueprint for building early-career researcher networks to advance NAM-based risk assessment
Source: NAM J. 2026 Aug 7;2:100124. doi: 10.1016/j.namjnl.2026.100124 (PMC13482515; doi:10.1016/j.namjnl.2026.100124)
Supplement: Supplementary file 5 [file mmc5.pdf]

Manual for ESR mobility from the ASPIS Academy Twinning program

Objective:
This manual stems from the ASPIS Twinning Program and aims to provide opportunities for Early Stage Researchers (ESRs) that participate in collaborative projects to visit and collaborate with laboratories across their network.

This manual is intended to guide ESRs in establishing a connection with institutions of their interest and assist in finding funding opportunities for these exchanges.

In the case that in-person exchanges are not feasible, online meetings between ESRs and researchers at their institution of interest should also be considered to allow ESRs to discuss specific techniques, methods, or collaborative ideas directly with potential host labs without committing to a full secondment.

Program details:

- **Duration of the exchange:** The duration of each exchange will be discussed and determined individually. It will depend on the specific needs of the student, the objectives of the visit, and the available budget. Students and their host PIs are encouraged to plan a feasible timeline that aligns with these factors.
- **Funding**: Students can explore available scholarship opportunities with the assistance of the program.

Step-by-Step Guide

1. Identify Your Target Institution

- **Research the available institutions**: Review the list of institutions involved in your project/consortium. Identify those with research areas that align with your interests and objectives.

- **Understand Their Expertise**: Gather information about the research focus and projects undertaken by your target institution.
    - **Tip**: Focus on labs with methodologies or technologies that would significantly benefit your own research work.

2. Justify Your Selection

- **Write a Statement of Purpose**: Clearly outline why you want to collaborate with the selected institution.
    - **Describe the Relevance**: Explain how the research or techniques from this lab align with your current work.
    - **Highlight the Benefits**: Articulate what specific skills or knowledge you aim to gain and how this experience will contribute to your growth as an early-stage researcher (e.g., PhD or Master's student).
    - **Consider Long-term Impact**: Mention how this collaboration might open future research or career opportunities.

3. Contact the Lab's Principal Investigator (PI)

Contact your own PI involved in the collaborative project and/or the project coordinator, as they can help you reach the responsible person at the host institution of your interest. Discuss your intentions for the secondment with your PI or coordinator so that the purpose of your exchange is clearly defined. This will ensure that, when they put you in touch with the host institution, the goals of your visit are well communicated. Prepare an introduction email to support this process and facilitate further communication.

4. Find Funding Opportunities

- **Local Funding Options**: To begin, students should look into potential funding sources available through their home institution or local organisations. Some options include:
    - **Contacting your institution's international relations or research support office**: These departments often have information on available grants, scholarships, or travel funds specifically for international internships or collaborative research. They can also provide guidance on applying for such opportunities.
    - **Institutional Partnerships**: Some universities have partnerships with specific labs or research centres and may offer funding for these collaborations. It's worth discussing with your institution's relevant offices to see if any targeted funding exists.
    - **Region-specific funding**: Many countries and regions offer funding specifically for international exchange programs. Look into national or regional scholarships that support academic mobility and international internships. Here are some country-specific options (more info in the Doctoral Mobility Funds and ERA-NET NEURON documents):
        - **Austria**: The Austrian Science Fund (FWF) offers mobility programs such as the Erwin Schrödinger Fellowships, which support postdoctoral researchers in gaining international experience.

- **Belgium**: The Research Foundation - Flanders (FWO) provides various grants, including the Junior Postdoctoral Fellowship, which encourages international mobility.
- **France**: The French National Research Agency (ANR) offers funding for international mobility through programs like the "Make Our Planet Great Again" initiative.
- **Finland**: The Research Council of Finland (AKA) funds international mobility through programs based on bilateral agreements, covering travel and living expenses for researchers. Applications for mobility funding are invited in August–September.
- **Germany**: The German Academic Exchange Service (DAAD) provides numerous scholarships and grants for researchers at all stages, including the Research Grants - Short-Term Grants for doctoral candidates and young researchers.
- **Italy**: The Farnesina (Ministry of Foreign Affairs) provides annual grants for the co-financing of Youth Exchange projects. The Ministry of Education, Universities and Research (MIUR) supports mobility through programs like the Rita Levi Montalcini Program, which targets young researchers.
- **Spain**: The Spanish Ministry of Science and Innovation offers various grants, such as the José Castillejo para jóvenes doctores and many others, which promote the mobility of early-stage researchers.
- **Switzerland**: The Swiss National Science Foundation (SNSF) provides funding for international mobility through programs like the Early Postdoc.Mobility fellowships.

- **EU Funding Programs**: The European Union provides several well-established funding programmes designed to support international mobility and research collaboration. Key options include:
  - **Erasmus+** (https://erasmus-plus.ec.europa.eu/): A highly flexible program offering grants to students, researchers, and staff for international exchanges, internships, and research projects within Europe. Erasmus+ supports not only mobility but also cooperation between academic institutions and private organisations.
  - **Marie Skłodowska-Curie Actions (MSCA)** (https://marie-sklodowska-curie-actions.ec.europa.eu/): MSCA supports researchers through mobility, training, and career development. It offers funding for doctoral candidates, early-stage researchers, and experienced researchers. Researchers involved in collaborative projects, could qualify for MSCA funding to support their exchange.
  - **Horizon Europe** (https://research-and-innovation.ec.europa.eu/funding/funding-opportunities/fellowships-and-individual-research-grants_en): While primarily focused on large-scale research collaborations, Horizon Europe also provides funding for mobility and international exchange in the context of EU research initiatives. Many EU-based labs and research centres may

be eligible for Horizon Europe grants, which can support ESR visits to partner labs.

  - **European Research Council (ERC) Grants** (https://erc.europa.eu/apply-grant): These grants are awarded to researchers of any nationality working in any field of research. They support frontier research and innovation.
  - **COST (European Cooperation in Science and Technology) Actions** (https://www.cost.eu/): COST supports networking and collaboration among researchers, including PhD students, through short-term scientific missions, training schools, and other mobility opportunities. While PhD students cannot initiate a COST Action, they can join existing ones by creating an e-COST profile and applying to participate in working groups.
  - **EUROTOX Early Career Exchange Program (ECEP)** (https://www.eurotox.com/ecep/): ECEP supports early career researchers in toxicology by funding international exchanges to gain new scientific techniques, regulatory knowledge, and collaborations. Five successful applicants receive up to €1000 for travel and accommodation. Eligible applicants are MSc, MD, or PhD students, or researchers within five years of their highest degree, based in Europe. Exchanges must occur in a country with an active EUROTOX member society (excluding Russia). Exchanges must take place between September 1, 2025, and April 30, 2026. Application deadline: April 30 2025. The ECEP program is planned to take place every year.

- **Tips for Funding Applications**: When applying for any of the above funding options, it’s important to frame the collaboration as beneficial to both the student and the host lab. Consider the following points to strengthen your application:
  - **Emphasise the collaborative nature of the visit**: Highlight how the exchange will promote scientific collaboration, joint research, and mutual learning between the ESR and the host institution. Funders are more likely to support initiatives that facilitate long-term partnerships and knowledge-sharing. If your project is a collaborative initiative supported by the EU, it’s beneficial to underscore the cooperation between institutions involved in various projects. Emphasise how the exchange will strengthen the existing network, enhance cross-institutional research, and contribute to shared scientific goals across the community.
  - **Describe the learning outcomes**: Articulate what you will gain from the exchange, including exposure to new research methodologies, expertise, and international networking opportunities. If you are enrolled in a PhD program, make the connection to your doctoral research, explaining how the collaboration will complement and advance your project. Highlight how the exchange will provide access to cutting-edge techniques, foster interdisciplinary collaboration, and enhance your academic and professional development as an ESR, all of which will contribute to the successful completion of your PhD and further your research career.
  - **Highlight potential for knowledge transfer:** Explain how the knowledge and skills acquired during the exchange will be shared with other students, researchers, and the home institution, contributing to broader scientific progress.

    - **Detail the impact on career development:** Show how the visit will advance the your research career, enhancing your skills, competencies, and future research opportunities.
- **Request Support from your project/institution**: Your project coordinators, supervisors, or host institution can be valuable allies in navigating the funding application process. If you need assistance finding scholarships or specific funding opportunities, or if you have questions about which options are best for your research exchange, consider the following:

    - **Seek advice** on available funding options, both regionally and internationally, from experienced colleagues or project coordinators.

    - **Ask for guidance** in identifying institutions that align best with your learning goals, especially if you have a specific technique or topic in mind but are unsure where it is being developed.

    - **Contact the grant development or research support office** at your home institution for help in tailoring your funding applications to meet eligibility criteria and maximise success.

    - **Request support** for the completion of required documentation or formal letters, including an official endorsement of your visit.

5. Organize Your Visit

- **Coordinate with the Host Institution**: Once funding is secured and the PI has agreed to host, work together to set a timeframe for your visit.
    - **Discuss Research Goals**: Define the specific techniques or knowledge areas you will focus on during the visit.
- **Prepare Travel and Accommodation Arrangements**: Coordinate with your host institution and explore options for accommodation near the lab.

6. Engage in Online Knowledge Exchange (Optional)

- **Request Online Sessions with Experts**: If a physical visit is not feasible or if additional knowledge is needed, ask about the possibility of online sessions with other labs.
    - **Identify Specific Techniques or Methods**: Clearly state what you hope to learn in these sessions.
- **Coordinate Scheduling**: Work with the expert to schedule sessions at a mutually convenient time.
- **Follow-Up**: After the session, send a thank-you email and, if relevant, summarize the key insights gained during the discussion.

7. Reporting and Feedback

- **Share Your Experience**: After your visit or online sessions, we encourage you to share your experience in a format that works best for you. This could be a short, informal talk with your PI and/or project coordinator, a few paragraphs

describing what you learned (especially if you've already prepared a report for your university), or even a fun news-style article for your project’s website.
